# Identifying subgroups of Chinese men who have sex with men based on sexual behavior and drug use patterns using a clustering analysis approach

**DOI:** 10.1186/s12889-025-22388-x

**Published:** 2025-04-10

**Authors:** Bingyang She, Jiajun Sun, Fang Lu, Siqi Lin, Yi Liu, Gaixia Li, Yawu Hu, Weiming Tang, Rayner Tan, Jason Ong, Shu Su, Lei Zhang

**Affiliations:** 1 Phase I clinical trial research ward, The Second Affiliated Hospital of Xi’an Jiaotong University, No.157 Xi Wu Road, Xi’an, 710004 Shaanxi Province PRC China; 2 China-Australia Joint Research Center for Infectious Diseases, School of Public Health, Xi’an Jiaotong University Health Science Center, Xi’an, 710061 Shaanxi China; 3 Melbourne Sexual Health Centre, Alfred Health, Melbourne, Australia; 4 School of Translational Medicine, Faculty of Medicine, Nursing and Health Sciences, Monash University, Melbourne, VIC Australia; 5 Department of Epidemiology and Biostatistics, The Second Affiliated Hospital of Chongqing Medical University, Chongqing, China; 6 Institute for Global Health and Infectious Diseases, University of North Carolina at Chapel Hill, Chapel Hill, NC USA; 7 Saw Swee Hock School of Public Health, National University of Singapore, National University Health System, Singapore City, Singapore

**Keywords:** Sexualized drug use, MSM, Cluster analysis, Sexual behavioral patterns, Sexual acts

## Abstract

**Introduction:**

Sexualized drug use (SDU) refers to using drugs before and during sex to enhance experiences, increasing high-risk behaviors, such as condomless sex, multiple sexual partners, and participation in group sex, among men who have sex with men (MSM). This study explores how SDU affects sexual behaviors in Chinese MSM.

**Methods:**

We collected demographics, sexual acts, drug use, and condom attitudes data among 890 MSM from across China via WeChat ads through community-based organizations from March 23 to April 22, 2022.Cluster analysis using Gower’s distance and hierarchical clustering explored differences in sexual acts among MSM who reported SDU in their last encounter and otherwise.

**Results:**

Cluster analysis categorized participants into three Clusters. Cluster 3 (*n* = 155) reported 100% SDU in their last sexual encounter (83.87% poppers use), whereas Clusters 1 (*n* = 581) and 2 (*n* = 154) reported none. Compared to other Clusters, Cluster 3 had significantly higher PrEP use (34.90% vs. 17.02% vs. 8.00%, *p* < 0.0001), more sexual acts over the past 12 months (35.80–61.30 vs. 31.30–56.10 and 4.37–21.22, *p* < 0.001), more regular (3.16 ± 4.37 vs. 2.27 ± 3.52 vs. 2.51 ± 2.53, *p* = 0.028) and casual partners (4.55 ± 6.55 vs. 2.48 ± 3.21 vs. 2.74 ± 3.66, *p* < 0.0001), more partners with STIs (8.39% vs. 3.79% vs. 3.90%, *p* = 0.029), and lower consistent condom use (48.53% vs. 59.41% vs. 72.28%, *p* < 0.0001). Cluster 1 had moderate frequency in all sexual acts except self-masturbation, which was most common in Cluster 2.

**Conclusion:**

SDU is a stratum for identifying MSM subgroups, and MSM who reported SDU demonstrated higher sexual risk behaviors and PrEP usage. Among those not practicing SDU, self-masturbation is a key behavioral indicator for subgrouping.

**Supplementary information:**

The online version contains supplementary material available at 10.1186/s12889-025-22388-x.

## Introduction

Sexualized drug use (SDU), which includes chemsex, is defined as the intentional engagement in substance use before or during sexual contact to enhance the experience and has emerged as a worldwide concern. The substances involved in SDU often include mephedrone, crystal methamphetamine, Gamma hydroxybutyrate/Gamma butyrolactone (GHB/GBL), cocaine, and ketamine [1–5]. Increasingly, the interplay between substance use and high-risk sexual behavior has led to public health challenges, particularly among men who have sex with men (MSM). SDU, alongside inherently other high-risk sexual practices, elevates the risk of sexually transmitted infections (STIs) in MSM [1, 6, 7]. The prevalence of SDU in MSM varies significantly across different countries and regions, ranging from 34 to 79% in developed nations such as the United States, United Kingdom, and Germany, whereas in China, prevalence ranges between 18 and 28% [8, 9].

SDU can profoundly affect cognitive functions and increase risk-taking sexual activity. Under the influence of drugs, MSM may engage in prolonged sexual sessions, potentially resulting in physical injuries or traumas [10]. Moreover, high-risk sexual behavior under the influence of SDU, like condomless sexual behavior, increases the spread of HIV and STIs [11]. Further, drug-enhanced sense of intimacy can lead individuals to engage in various sexual acts during a single encounter and facilitate group sex, increasing the risk of HIV/STI exposure [9, 11, 12].

Although previous studies have explored the link between SDU and high-risk sexual behaviors such as condomless intercourse, group sex, and overall sexual risk-taking, the specific patterns and frequencies of individual sexual acts, like insertive and receptive anal sex, rimming, and oral sex, remain underexplored [1, 13–21]. The specific patterns and frequencies of individual sexual acts (e.g., insertive anal sex, receptive anal sex, rimming, oral sex) remain underexplored. For example, only a few studies have suggested a potential association between the frequency of rimming and the use of specific drugs (e.g., poppers, methamphetamine, GHB, ecstasy/MDMA) [7, 22, 23]. Consequently, how SDU influences the frequency and combination of these distinct sexual behaviors in MSM remains unclear, leaving a significant gap in our understanding of the relationships between SDU and sexual behavioral patterns. Addressing this knowledge gap is crucial for developing more targeted interventions and prevention strategies.

Conventional analytical approaches face challenges in identifying the subtle differences across various subpopulations in MSM due to the preference and complex nature of their sexual behaviors. To overcome this, we used Cluster analysis, a machine learning technique, to simplify the data and uncover underlying patterns. Cluster analysis is widely used in behavioral studies to identify subpopulations [24–27], making it an appropriate approach for analyzing SDU and related sexual behaviors in MSM. This study aims to use Cluster analysis to identify MSM subpopulations according to their SDU behaviors and compare the differences in their sexual behavioral patterns across identified Clusters. This approach may provide evidence for targeted intervention of high-risk sexual behavior and drug use during sexual encounters in MSM.

## Methods

### Study design and participants recruited

Our study was an anonymous cross-sectional survey across China from March 23 to April 22, 2022, through community organizations and social groups. We recruited MSM nationally through advertisements on the WeChat platforms of six major gay service groups including Zhuhai Xutong Volunteer Service Center, Qingdao Qingtong Anti-AIDS Volunteer Service Center, Nanjing Xingyou Volunteer Service Center, Jinan Rainbow, Beijing LGBT + and Positive Peers Group, which are dedicated to HIV and STI education and intervention. The recruitment process provided detailed study information which included a general description of the project and addresses of participating health service stations and Centers for Disease Control and Prevention (CDCs) and offered cash incentives for participation. Eligible participants who consented to participate were directed to complete a detailed questionnaire on sexual behavior, substance use and recent sexual activities with men at Weng Juan Xing (www.wjx.cn). To be eligible for participation in the questionnaire, individuals had to meet three criteria: (1) biologically assigned as male at birth; (2) have a history of male-to-male anal sex; and (3) be between the ages of 18 and 70, as sexual activity tends to be lower in individuals over 70 due to physiological factors.

### Measures

At the beginning of our study, participants were asked to provide information about their sociodemographic characteristics, including gender, age, income, marital status, educational qualifications, and self-perceived gender identity. Regarding their sexual activity, the questionnaire surveyed the number of days since their last sexual acts and the sequence of their most recent sexual encounter. A significant part of the questionnaire focused on understanding drug use during their most recent sexual encounter. Participants were asked the question, ‘Did you use drugs during the last time you had sex with a man?’ and were given a list of substances to choose from, including Rush, Crystal Meth, and Ketamine, etc.

We collected data on the frequency of sexual encounters with both regular and casual male partners as well as the frequency of condom use over the past six months. We also recorded the number of days since the most recent sexual act, which ranged from kissing to more intimate acts such as insertive and receptive oral sex, anal sex, rimming, and masturbation (self, for a partner, by a partner). For full details on measure assessment, please refer to the Appendix.

### Definition of SDU

We defined SDU as the use of substances before or during a sexual encounter. These substances include, but are not limited to, Poppers, Ketamine, Ecstasy (MDMA), and GHB/GBL.

### Definition of regular and casual sexual partnerships

Regular sexual partners were defined as individuals in a committed and lasting romantic or sexual relationship for more than three months. A relationship is classified as ‘regular’ only after exceeding this duration, regardless of the initial intention for a long-term relationship. Casual sexual partners were defined as individuals with whom sexual relationships lasted three months or less, including male sex workers.

### Definition of High-risk behaviors

High-risk behaviors in this study include condomless sex, engaging in sexual activities with multiple partners, group sex, and the use of substances that may impair judgment or lead to unsafe sexual practices. These behaviors are associated with an increased risk of acquiring or transmitting sexually transmitted infections (STIs), including HIV [28].

### Clustering analysis

We selected 11 sexual behavioral indicators for clustering analysis, including the number of days since the participant’s last sexual encounter involving sexual acts such as kissing, insertive or receptive oral sex, insertive or receptive anal sex, rimming, masturbation (self, with a partner, or by a partner), and drug use during the last sexual encounter (SDU). Given the combination of continuous variables (e.g., the number of days since the participant’s last sexual acts) and binary categorical variables (e.g., SDU), we employed the Partitioning Around Medoids (PAM) algorithm using Gower’s distance to handle these mixed data types and the silhouette coefficient to determine the optimal number of Clusters. Multidimensional scaling (MDS) was used to standardize both categorical and continuous variables [29]. PAM clustering analysis separated the population into two groups: those with SDU and those without. Heterogeneity within the non-SDU group prompted us to investigate further potential subgroups in this group using a hierarchical clustering approach based on sexual behavior frequency. The combined PAM and hierarchical clustering enabled us to account for both participants’ drug use and sexual behavioral patterns at two different levels. We used t-SNE for dimensionality reduction and visualization of the clustering structure in two dimensions. The visualization helped confirm the existence of Clusters and sub-Clusters.

### Estimating the number of sexual acts over the past 12 months

Our method for estimating the frequency of sexual acts in MSM, including handling missing data, has been validated in a previous study, which used similar methods to quantify the sexual behaviors in MSM populations [29]. We employed a log-normal distribution fitting method to estimate the frequency of various sexual behaviors, as the log-normal distribution effectively simulates the distribution of complex human behaviors such as sexual acts. Based on the log-normal distribution fitting, we estimated the number of different sexual acts based on the number of days since the last sexual acts that were reported [30, 31]. We then calibrated the simulated frequency to the observed accumulated frequency of sexual acts in the participants. This calibration enabled us to estimate the distribution of the number of sexual acts by their sexual act types even in the absence of empirical data. The calibration was considered successful when the difference (defined as Mean Square Error (MSE)) between the simulated and observed data was minimized:

$$\:MSE=\frac{1}{n}\sum\:_{i=1}^{n}{\left({\stackrel{\prime }{y}}_{i}-{y}_{i}\right)}^{2}$$

Where, ${\mathop y\limits^\prime _i} \sim LN$($\:{\mu\:}_{i,}{\delta\:}_{i}$) represents the log-normal distribution curve of the number of days of the last sex behaviors on type $\:i$, mean $\:{\mu\:}_{i}$ and variance $\:{\delta\:}_{i}$, and $\:{\stackrel{\prime }{y}}_{i}$ represents a randomly generated bootstrap datasets corresponding to the original datasets of sexual acts. The Mean Square Error (MSE) was used to compute the mean value of the sum of the squares of errors between the estimated data point and the bootstrapped data point. Bootstrapped data was generated by resampling the original data to create a dataset of similar dimensions as the fitted data. We repeated the estimated process 400 times and selected the curve with the smallest MSE value as the optimized curve. The best 50 fittings were chosen to establish a 95% confidence interval. After estimating the curves, we obtained their log-probability density function values. These probabilities were then converted into weights for days 1 to 365. The weights were used to estimate the number of sexual acts over the last 12 months. This allowed us to calculate the weighted mean number of sexual acts over last 12 months. The entire optimization and fitting data process was conducted using MATLAB R2020a.

### Statistical analysis

Categorical variables in this study were described using numbers and percentages, and the chi-square test was used to compare differences between groups. For normally distributed continuous variables, mean ± standard deviation was used to present the data, and ANOVA (analysis of variance) was performed for comparison. Non-normally distributed continuous variables were reported as median and interquartile range, and differences between groups were assessed using the Wilcoxon rank-sum test.

### Result

#### Clustering participants by sexual and drug-use behaviors

**Fig. 1:**
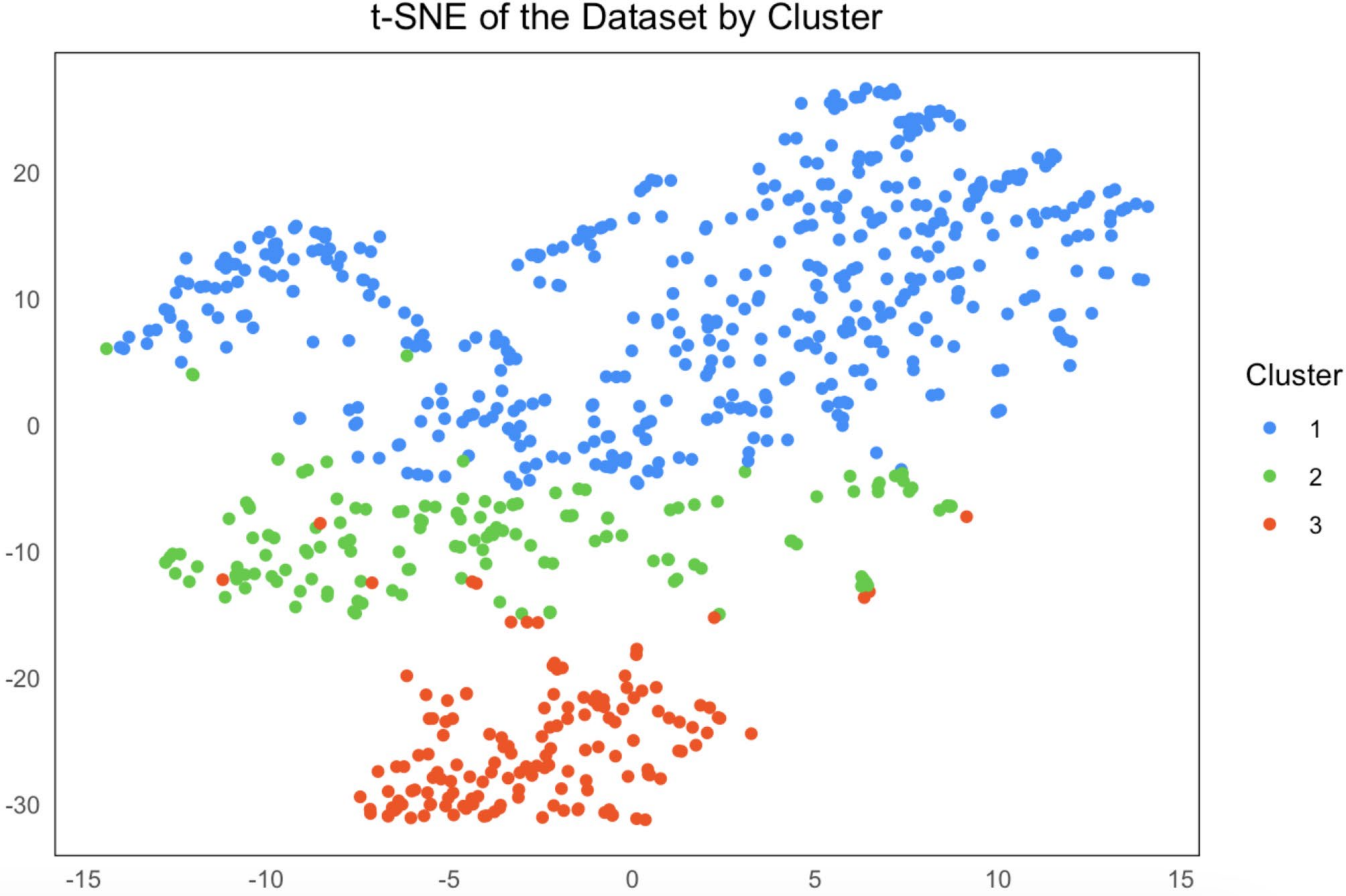
Reduced dimensional scatter plot of three clustering results

Our study initially surveyed 1,034 individuals, excluding 144 due to lack of consent (*n* = 100), those who were not within the required age range (*n* = 42), or duplicate responses (*n* = 2). Ultimately, 890 participants were included in the analysis (Figure S1). The first step of the clustering analysis categorized participants into two Clusters: those whose most recent sexual encounter reported SDU (*n* = 155) and those who did not report SDU (*n* = 735) (Figure S2). Subsequently, the second step of the clustering analysis further divided the non-SDU in their last sexual encounter Cluster into two subgroups based on their sexual activity frequency. Cluster 1 (*n* = 581) consisted of individuals with higher sexual activity frequency, while Cluster 2 (*n* = 154) was characterized by lower sexual activity frequency. This resulted in a total of three distinct Clusters. (Cluster 1, *n* = 581; Cluster 2, *n* = 154; Cluster 3, *n* = 155) (Fig. 1, S3).

#### Differences in demographic characteristics across clusters

The majority of the study participants were single (88.99%), predominantly held a college or bachelor’s degree (73.37%) and had an income range of RMB 5001–8000 (26.07%). The average age of participants was 28.14 ± 7.37 years. About 17.42% of the participants reported engagement in SDU in their last sexual encounter. (Table 1)

**Table 1:** Demographic and behavioral characteristics of study participants, stratified by clusters

Category	Overall (*N* = 890)	Cluster1 (*N* = 581)	Cluster2 (*N* = 154)	Cluster3 (*N* = 155)	Chi^2^/F	*P*-value
Age (years)	28.14 ± 7.37	28.15 ± 7.22	27.85 ± 8.06	28.40 ± 7.27	0.215	0.806
**Marital Status**					6.591	0.159
Single	792 (88.99%)	525 (90.36%)	132 (85.71%)	135 (87.1%)		
Engaged or Married	54 (6.07%)	30 (5.16%)	10 (6.49%)	14 (9.03%)		
Separated or Divorced or Widowed	44 (4.94%)	26 (4.48%)	12 (7.79%)	6 (3.87%)		
**Education level**					8.19	0.085
High school or below	120 (13.48%)	89 (15.32%)	19 (12.34%)	12 (7.74%)		
College/Bachelors	653 (73.37%)	421 (72.46%)	116 (75.32%)	116 (74.84%)		
Masters or above	117 (13.15%)	71 (12.22%)	19 (12.34%)	27 (17.42%)		
**Personal monthly income (RMB)**					4.708	0.788
< 1500	106 (11.91%)	62 (10.67%)	23 (14.94%)	21 (13.55%)		
1500–3000	112 (12.58%)	73 (12.56%)	21 (13.64%)	18 (11.61%)		
3001–5000	225 (25.28%)	152 (26.16%)	39 (25.32%)	34 (21.94%)		
5001–8000	232 (26.07%)	149 (25.65%)	38 (24.68%)	45 (29.03%)		
> 8001	215 (24.16%)	145 (24.96%)	33 (21.43%)	37 (23.87%)		
**Use drugs during last sexual encounter**						
Yes	155 (17.42%)	0 (0%)	0 (0%)	155 (100%)	890	<0.0001*
No	735 (82.58%)	581 (100%)	154 (100%)	0 (0%)		
**Drug Types**						
Poppers	130 (83.87%)	0 (0%)	0 (0%)	130 (83.87%)	890	<0.0001*
DMT	1 (0.65%)	0 (0%)	0 (0%)	1 (0.65%)		
Triazolam	1 (0.65%)	0 (0%)	0 (0%)	1 (0.65%)		
Others	23 (14.84%)	0 (0%)	0 (0%)	23 (14.84%)		
Crystal Meth	0 (0%)	0 (0%)	0 (0%)	0 (0%)		
Ketamine	0 (0%)	0 (0%)	0 (0%)	0 (0%)		
Ecstasy	0 (0%)	0 (0%)	0 (0%)	0 (0%)		
Ya ba pills	0 (0%)	0 (0%)	0 (0%)	0 (0%)		
Mixing drugs	0 (0%)	0 (0%)	0 (0%)	0 (0%)		
GHB	0 (0%)	0 (0%)	0 (0%)	0 (0%)		
Monkey dust	0 (0%)	0 (0%)	0 (0%)	0 (0%)		

Note: DMT: N, N-Dimethyltryptamine; GHB: Gamma-Hydroxybutyrate

While comparing the demographic characteristics of the participants, we observed that all participants who reported SDU in their last sexual encounter were exclusively found in Cluster 3 (100% versus 0% in both Cluster 1 and Cluster 2, chi-2 test, *p* < 0.001). In Cluster 3, the most common drug used was poppers (130 participants, 83.87%), followed by other drugs (23 participants, 14.84%). DMT and Triazolam were each used by 1 participant (0.65%). Apart from the engagement in SDU in their last sexual encounter, there were no statistically significant differences among the Clusters in terms of age, marital status, education level, and personal monthly income (all *p* > 0.05). (Table 1)

#### Difference in SDU patterns across clusters in last 12 months

Of the three MSM Clusters identified, Poppers (34.25% vs. 30.52% vs. 52.26%) and erectile dysfunction medications (e.g., Viagra) (16.53% vs. 12.34% vs. 18.71%) were the most commonly reported SDU in last 12 months. Cluster 3 exhibited the highest frequency of drug use, with the “more than half the time” usage rate significantly higher than the other two Clusters for Poppers (12.90% vs. 4.55% vs. 4.82%, chi-squared test, *p* = 0.0022) and erectile dysfunction medications (2.58% vs. 1.20% vs. 1.95%, chi-squared test, *p* = 0.0211). Cluster 1 reported low-frequency drug use, with a small proportion of participants using “other” substances (2.41%), heroin (1.38%), marijuana (1.38%), ketamine (1.55%), ecstasy (1.55%), and GHB/GBL (1.38%). Cluster 2 exhibited the lowest drug use frequency, demonstrating relatively conservative patterns in the use of various substances. (Table S1)

#### Difference in frequency of sexual acts across clusters

The median number of days since last sexual activity in the study population ranged from 3 to 365 days. Self-masturbation had the shortest duration at 3 days, while rimming had the longest duration at 365 days. The estimated number of sexual acts over the past 12 months was 88.80 (95% CI 59.50-153.18) for self-masturbation, 43.24 (95% CI 41.00-50.35) for insertive anal sex, 38.16 (95% CI 31.19–48.23) for receptive anal sex, 49.38 (95% CI 52.07–73.72) for insertive oral sex, 46.97 (95% CI 51.44–59.77) for receptive oral sex, 34.90 (95% CI 25.03–45.18) for rimming, and 34.99 (95% CI 25.57–46.39) for being rimmed. (Table S2-3)

When comparing the clustering results of the three Clusters, Cluster 3 had a significantly lower median number of days since last sexual activity for sexual acts with a partner, compared to Cluster 1 and Cluster 2 (median days range: 5–10 versus 7–19 and 90–200, Wilcoxon, *p* < 0.001). Consistently, Cluster 3 had a significantly higher average number of sexual acts except for self-masturbation (average number of acts: 88.80 (95% CI 65.33, 147.79) versus 82.94 (95% CI 62.81, 119.90) and 118.52 (95% CI 70.32, 175.75), Wilcoxon, *p* < 0.001) over the past 12 months, compared to Cluster 1 and Cluster 2 (average acts range: 35.80–61.30 versus 31.30–56.10 and 4.37–21.22, Wilcoxon, *p* < 0.001).

**Fig. 2:**
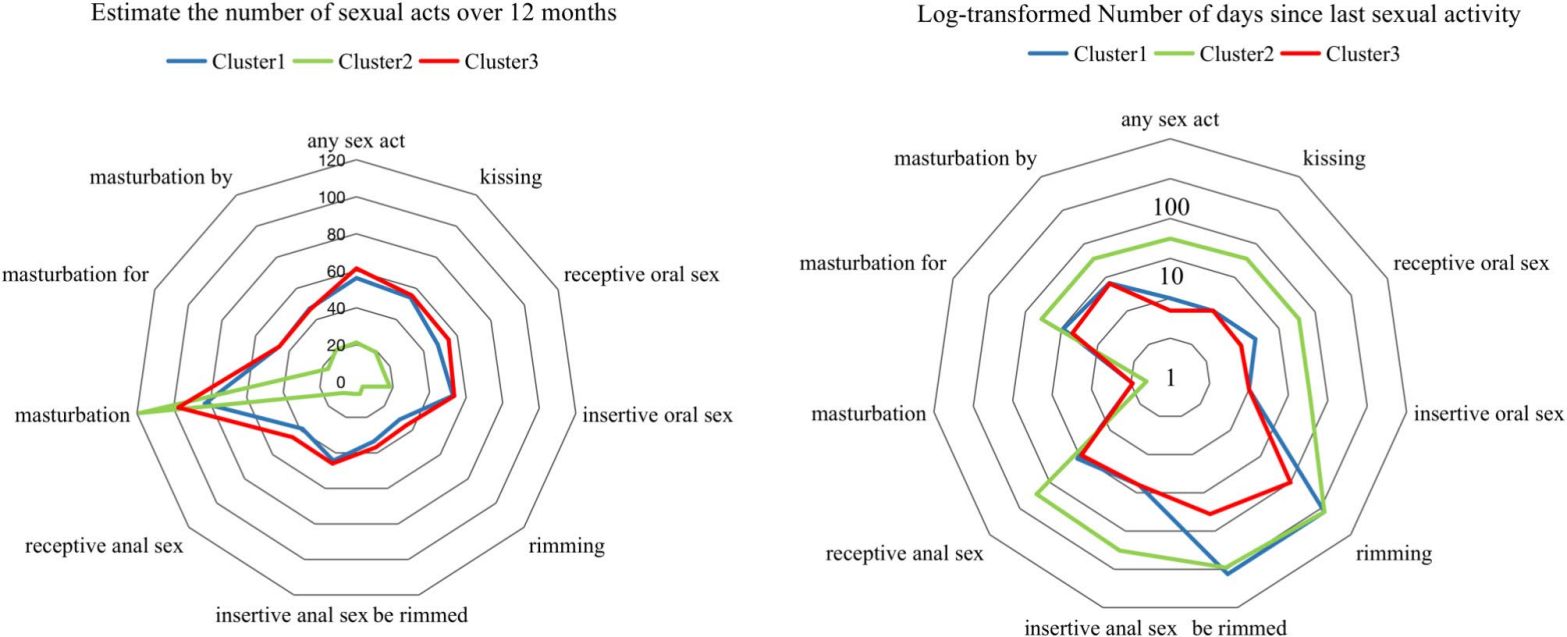
Radar plot demonstrating the number of days since the most recent sexual encounter and the estimated frequency of sexual acts over the past twelve months

The difference in sexual behavior frequency between Cluster 1 and Cluster 2 was particularly notable, and neither group reported SDU (sexualized drug use) in their last sexual encounter. Cluster 1 engaged in a variety of sexual acts, including insertive and receptive oral and anal sex, with a moderate level of sexual activity, averaging 35.80 to 61.30 sexual acts over the past year. In contrast, Cluster 2 had much lower levels of sexual activity, with self-masturbation being the predominant sexual act. Sexual acts with partners were rare in Cluster 2, with an average of only 4.37 to 21.22 acts, indicating that sexual activity was primarily limited to self-masturbation, with minimal engagement with partners. Importantly, neither Cluster 1 nor Cluster 2 participants reported SDU in their last sexual encounter, in stark contrast to Cluster 3. The radar chart clearly displayed the differences in clustering results among the three groups. (Fig. 2)

#### Difference in sexual history, PrEP use, and STI testing across clusters

When comparing the three Clusters, several key differences were observed. Notably, Cluster 1 and Cluster 2 members were not entirely free from SDU. SDU during sexual encounters prior to the past year was significantly more common in Cluster 3 (94.84% vs. 41.83% vs. 43.51%, chi-2 test, *p* < 0.0001). Cluster 3 had the significantly highest proportion of participants who had ever taken PrEP (34.90% vs. 17.02% vs. 8.00%, chi-2 test, *p* < 0.0001) and had the significantly highest willingness to take it in the future (64.95% vs. 55.98% vs. 47.10%, chi-2 test, *p* < 0.0001). In the last six months, Cluster 3 also had significantly more casual male sex partners (4.55 ± 6.55 vs. 2.48 ± 3.21 vs. 2.74 ± 3.66, F = 12.49, *p* < 0.0001) and regular male sex partners (3.16 ± 4.37 vs. 2.27 ± 3.52 vs. 2.51 ± 2.53, F = 3.58, *p* = 0.028) and reported the significantly highest percentage of having sexual partners infected with an STI (8.39% vs. 3.79% vs. 3.90%, chi-2 test, *p* = 0.029). Additionally, Cluster 3 had the significantly highest frequency of participation in group sex (29.03% vs. 7.84% vs. 8.44%, chi-2 test, *p* < 0.0001) and the significantly lowest rate of always using condoms during anal sex with casual male partners (48.53% vs. 59.41% vs. 72.28%, chi-2 test, *p* < 0.0001). Despite the lack of statistical significance, Cluster 3 had a higher proportion of participants reporting a positive HIV test result (3.92% vs. 3.10% vs. 2.80%, chi-2 test, *p* = 0.2) and recent STI diagnoses other than HIV (9.03% vs. 5.85% vs. 5.84%, chi-2 test, *p* = 0.334) compared to the other Clusters. (Table 2)

**Table 2:** Sexual history, PrEP use, and STI testing among study participants, stratified by clusters

Category	Cluster 1 (*N* = 581)	Cluster2 (*N* = 154)	Cluster3 (*N* = 155)	Chi^2^/F	*P*-value
**Have you ever taken PrEP?**				38.61	< 0.0001
Yes	96 (17.02%)	12 (8.00%)	52 (34.90%)		
No	468 (82.98%)	138 (92.00%)	97 (65.10%)		
**If you haven’t taken PrEP yet**,** would you want to?**				43.75	< 0.0001
Yes	262 (55.98%)	65 (47.10%)	63 (64.95%)		
No	206 (44.02%)	73 (52.90%)	34 (35.05%)		
**What was the result of your last HIV test?**				8.6	0.2
Negative	514 (93.62%)	136 (95.10%)	145 (94.77%)		
Positive	17 (3.10%)	4 (2.80%)	6 (3.92%)		
Prefer not to say	18 (3.28%)	3 (2.10%)	2 (1.31%)		
**Have you been diagnosed with an STD other than HIV in the past 6 months?**				2.16	0.334
Yes	34 (5.85%)	9 (5.84%)	14 (9.03%)		
No	547 (94.15%)	145 (94.16%)	141 (90.97%)		
**Have you had any sexual partners infected with an STD in the last 6 months?**				10.79	0.029
Yes	22 (3.79)	6 (3.90%)	13 (8.39%)		
No	387 (66.61)	103 (66.88%)	85 (54.84%)		
Not sure	172 (29.6)	45 (29.22%)	57 (36.77%)		
**Have you ever used drugs in your sexual encounters prior to the past year?**				142.24	< 0.0001
Yes	243 (41.83)	67 (43.51%)	147 (94.84%)		
No	338 (58.18%)	87 (56.49%)	8 (5.16%)		
**The role assumed during anal sex with another man in the last six months**				12.08	0.017
Insertive	260 (44.75%)	60 (38.96%)	53 (34.19%)		
Receptive	222 (38.21%)	74 (48.05%)	65 (41.94%)		
Both	99 (17.04%)	20 (12.99%)	37 (23.87%)		
**The frequency of participation in group sex (with two or more people) in the last six months**				37.98	< 0.0001
Never	502 (86.40%)	141 (91.56%)	109 (70.32%)		
Occasionally	75 (12.91%)	13 (8.44%)	40 (25.81%)		
Often	4 (0.69%)	0 (0)	5 (3.23%)		
Always	0 (0)	0 (0)	1 (0.65%)		
**Frequency of condom use during anal sex with casual male partners in the last 6 months**				28.75	< 0.0001
Never	39 (7.91%)	9 (7.83%)	12 (8.70%)		
Occasionally	98 (19.88%)	13 (11.30%)	33 (23.91%)		
Often	112 (22.72%)	19 (16.52%)	38 (27.54%)		
Always	244 (49.49%)	74 (64.35%)	55 (39.86%)		
**Frequency of condom use during anal sex with casual male partners in the last 6 months**				40.08	< 0.0001
Never	15 (3.71%)	3 (2.97%)	4 (2.94%)		
Occasionally	55 (13.61%)	7 (6.93%)	25 (18.38%)		
Often	94 (23.27%)	18 (17.82%)	41 (30.15%)		
Always	240 (59.41%)	73 (72.28%)	66 (48.53%)		
**Number of casual male sex partners in the last 6 months**	2.74 ± 3.66	2.48 ± 3.21	4.55 ± 6.55	12.49	< 0.0001
**Number of regular male sex partners in the last 6 months**	2.51 ± 2.53	2.27 ± 3.52	3.16 ± 4.37	3.58	0.028

Cluster 1 participants were more likely to have insertive anal sex (44.75% vs. 38.96% vs. 34.19%, chi-2 test, *p* = 0.017). Cluster 2 participants were more likely to have receptive anal sex (48.05% vs. 38.21% vs. 41.94%, chi-2 test, *p* = 0.017). Cluster 3 participants were more likely to assume both roles during anal sex in the last six months (23.87% vs. 17.04% vs. 12.99%, chi-2 test, *p* = 0.017) compared to other Clusters. (Table 2)

## Discussion

Among 890 Chinese MSM, we identified three distinct Clusters, finding that SDU in last sexual encounter was a significant criterion for differentiating sexual behavioral patterns. Cluster 3 all reported in SDU in last sexual encounter, while Cluster 1 and Cluster 2 did not. A key finding is that although Cluster 1 and Cluster 3 exhibited almost identical frequencies across various sexual acts—including oral sex, anal sex, and rimming—their risk profiles differed significantly. Cluster 3 exhibited significantly higher sexual risk behaviors, including having more casual and regular partners, lower consistent condom use, more frequent participation in group sex, and a higher proportion of partners with an STI in the past six months. The primary distinction between these two Clusters was SDU in last sexual encounter in Cluster 3, which seems to be the driving factor behind the elevated risk behaviors. In contrast, while Cluster 1 reported similar sexual act frequencies, it demonstrated fewer risk behaviors, such as more consistent condom use and fewer sexual partner with STIs. On the other hand, Cluster 2 primarily engaged in self-masturbation, had the least frequent sexual acts, and exhibited the lowest-risk sexual practices, such as the least participation in group sex, the highest adherence to condom use, the fewest regular and casual sexual partners, the lowest rates of STDs, and the highest proportion of individuals who had tested for HIV. In the past 12 months, sexual behavioral patterns among the three MSM Clusters showed distinct variations. Cluster 3 had the highest frequency of sexual encounters involving substance use, followed by Cluster 1 and Cluster 2, with the latter exhibiting the lowest frequency.

All participants in Cluster 3 reported drug use, with the majority using poppers (83.87%). This finding contrasts with studies from other countries, which indicate a higher prevalence of cannabis, GHB/GBL, ketamine, and mephedrone use [32–36]. The lower rate of these drugs observed in this Cluster may be influenced by Chinese policies that have effectively curtailed the use of these drugs among MSM populations [37, 38]. Cluster 3 also showed the highest frequency of all types of sexual acts over 12 months. This contradicts a longitudinal study that showed a decline in all forms of anal sex among SDU participants over time [39]. This discrepancy may be because our study participants primarily used poppers, whereas the longitudinal study focused on MSM using more addictive drugs. Long-term use of such drugs may impair physical capacity, reducing the frequency of sexual acts. Our findings are consistent with a qualitative study where users reported that drug use enhanced their sexual experience and performance, which may psychologically increase the frequency of sexual encounters [40]. Additionally, 34.9% of participants in Cluster 3 reported had ever taken PrEP, which is slightly higher than the prevalence rates observed in other regions. Previous studies have indicated that MSM who had ever taken PrEP in China typically ranges from 19.5 to 24.7% [28, 41]. However, as noted in earlier research, PrEP use may contribute to lower condom usage, potentially increasing the risk of other STIs [9, 13, 15, 42–44].

Our findings in China mirror global trends where SDU is associated with higher-risk sexual behaviors in MSM populations. For example, studies in the U.S. and Europe have shown that MSM who use poppers, GHB/GBL, and methamphetamine engage in more casual partners, group sex, and lower condom use [1, 7, 45, 46]. The strong association between SDU and high-risk sexual behavior is evident across different cultural and regulatory contexts, suggesting that public health strategies need to adapt to global trends while considering local policies and cultural norms. Therefore, interventions for this Cluster should focus on harm reduction strategies, particularly in relation to SDU. Clear educational messaging is needed to emphasize reducing sexual risks by consistently using condoms, even while on PrEP, and promoting waiting until sober before engaging in sex to reduce impulsive risk-taking. Furthermore, efforts should be made to ensure easy access to condoms and lubricants in high-risk settings, such as MSM-friendly venues, clubs, and group sex parties. Event organizers in these environments should be encouraged to proactively provide safety supplies and promote safer sex practices. Although PrEP usage in this Cluster is relatively high, it is crucial to reinforce the importance of combining PrEP with condom use to prevent STIs beyond HIV.

Cluster 1 includes approximately 40% of members who had previously engaged in SDU but not recently. This group demonstrates high sexual activity with moderate numbers of sexual partners (3–5), primarily engaging in one-on-one sexual encounters, with little group sex participation. Condom use is inconsistent, with some members reporting occasional use. To distinguish Cluster 1 from Cluster 3, the key differences are sexual activity frequency and SDU involvement. Both Clusters have high sexual activity, but Cluster 3 is marked by more frequent SDU and more sexual partners. For Cluster 1, public health interventions should focus on promoting consistent condom use by offering regular health education programs that emphasize the importance of using condoms with both regular and casual partners. Additionally, ensuring access to condoms, oral condoms, and lubricants through both online and offline distribution channels is crucial. Targeted education on risk communication in one-on-one relationships should be provided, helping this Cluster understand that even in stable partnerships, maintaining proper safety measures is critical.

Cluster 2 consists of participants with more conservative sexual behaviors, fewer sexual partners, and a higher focus on self-masturbation. Although 40% had engaged in SDU in the past, none reported recent use. Their sexual acts are infrequent, and they exhibit high safety awareness, consistent condom use. This Cluster may be opaquer and experience significant social isolation and psychological stress [37, 38].To distinguish Cluster 1 and Cluster 2, key differentiating factors include lower sexual activity frequency and fewer sexual partners for Cluster 2. In addition, participants of Cluster 2 may show higher activity on social media platforms, which could serve as an effective method for identification. Therefore, public health interventions for this Cluster should include mental health support providing anonymous psychological counseling services to enable them to deal with sexual identity crisis and social stigma. In addition, a mobile app could be developed to help them anonymously locate nearby sexual health clinics and services, enhancing access to essential health resources.

Cluster 3 exhibited the highest frequency of Poppers and erectile dysfunction medication (e.g., Viagra) use, with a notably higher proportion reporting frequent drug use. Given that Cluster 3 participants are more likely to engage in stimulant use and Viagra consumption, a strategic intervention would focus on controlling access to these substances. The low frequency of illicit drug use in Cluster 3, likely influenced by national policies and legal restrictions, suggests that strengthening regulations surrounding the availability of stimulants and increasing education on their risks could be an effective measure for this group.

Our study revealed that self-masturbation was the most common sexual act among participants, occurring 88.80 times over 12 months, whereas being rimmed and rimming were the least frequent, with 34.90 times respectively. This finding is consistent with previous research, which suggested self-masturbation as the most prevalent and rimming as the least practiced sexual act [47]. Following self-masturbation, kissing also emerged as a commonly practice sexual act with 50.20 times over 12 months, and this finding is aligned with the widely perceived significance of kissing in intimate relationships [48].

Our study has several limitations. First, the questionnaire design did not distinguish between participants who refused to answer and those who genuinely did not engage in the behavior. As a result, we could not determine how many participants selected ‘no occurrence’ versus those who refused to respond. To address this, we used available data and log-normal distribution fittings to impute the missing values, providing a more robust estimate and reducing bias from missing data. Second, our study may misclassify habitual SDU users who did not report in SDU during their most recent sexual encounter; this was due to most participants not reporting frequency data despite having a history of SDU. Third, self-reporting could lead to recall or social desirability biases, challenging data authenticity. Fourth, the recruitment method used in this study primarily reached MSM who are actively engaged with sexual health services or community organizations, which may introduce potential selection bias. MSM who are more involved in health-focused activities or community initiatives may be overrepresented. This bias may limit the generalizability of the study’s findings to the broader MSM population. Further, the reliance on online recruitment through WeChat and community groups may exclude MSM who are less active on these platforms. Future research should focus on including MSM who are less connected to these networks to ensure a more comprehensive understanding of the population’s behaviors and risks. Fifth, our analysis used an unsupervised machine learning during clustering. To date, unsupervised models can be difficult to interpret due to the lack of predefined labels, making results highly dependent on the chosen algorithm and parameters, which introduces subjectivity. Additionally, clustering results are sensitive to the choice of algorithm (e.g., PAM vs. hierarchical clustering) and distance metrics (e.g., Gower’s distance), potentially leading to different Cluster classifications. Noise or outliers in the dataset may skew results and affect the interpretation of behavioral patterns. Sixth, we acknowledge that t-SNE results are not directly interpretable as clustering outcomes. Therefore, we included an interpretation of Fig. 1 in the Results section to clarify how the t-SNE visualization corresponds to the identified Clusters. Seventh, despite our efforts to clearly define regular and casual partners in the questionnaire, some participants may have mistakenly classified the same partner under both categories (e.g., a participant in a ‘stable’ relationship for less than three months may have counted the same partner in both responses). This potential misclassification could have introduced minor bias in the findings. Lastly, while our clustering analysis identified three distinct groups, it is unclear whether these Clusters can be reliably distinguished for public health interventions. Further research is needed to establish practical methods for recognizing and targeting these groups in public health settings.

## Conclusions

Using Cluster analysis, we identified three MSM subgroups with varying levels of high-risk sexual behavior. Cluster 3, involving MSM who engaged in SDU, showed the highest sexual risk and requires harm reduction strategies, including PrEP, condom promotion, and safer sex education. Cluster 1, while not reporting SDU, still exhibited higher-risk behaviors than Cluster 2 and would benefit from education on consistent condom use in relationships. Cluster 2, with the lowest risk, primarily engaged in self-masturbation and may need mental health support for social isolation. Future research should explore SDU’s long-term effects and targeted STI prevention strategies.

## Glossary

1. **MSM (Men Who Have Sex with Men)** Refers to men who engage in sexual activity with other men, regardless of their sexual orientation. This term includes all men who have sex with men, whether they identify as gay, bisexual, or otherwise.
2. **SDU (Sexualized Drug Use)** The use of drugs before or during sexual activity to enhance the experience. SDU is often associated with riskier sexual behaviors, such as unprotected sex or group sex, increasing the risk of HIV and sexually transmitted infections (STIs).
3. **PrEP (Pre-exposure Prophylaxis)** A preventive treatment for HIV where people at high risk of infection take antiretroviral medication daily to reduce the risk of contracting HIV.
4. **STI (Sexually Transmitted Infections)** Infections transmitted through sexual contact, such as gonorrhea, syphilis, chlamydia, and HIV.
5. **Mean Square Error (MSE)** A measure of the average squared difference between estimated values and actual values. It is used to evaluate the accuracy of the model used to estimate sexual behavior frequencies in the study.
6. **Gamma Hydroxybutyrate/Gamma Butyrolactone (GHB/GBL)** GHB and GBL are substances often used recreationally, sometimes referred to as “club drugs.” GHB is a central nervous system depressant, commonly used in small doses for its euphoric and sedative effects. GBL is a prodrug, meaning it is converted into GHB once ingested. Both substances are frequently associated with increased sexual arousal and disinhibition, and are sometimes used during sexual encounters, which may heighten the risk of unsafe sexual practices. Overuse can lead to loss of consciousness, respiratory issues, or overdose.
7. **Partitioning Around Medoids (PAM)** PAM is a clustering algorithm that identifies representative objects, or medoids, from a dataset. These medoids are used to group similar objects together, based on a chosen distance metric. Unlike k-means, PAM works well with non-Euclidean distances and is robust to outliers. In this study, Gower’s distance was used, which is suitable for mixed data types (continuous and categorical variables).

## Electronic supplementary material

Below is the link to the electronic supplementary material.

Supplementary Material 1

Supplementary Material 2

## Data Availability

The datasets used and/or analyzed during the current study are available from the corresponding author on reasonable request.

## Declarations

### Questionnaire Design

The questionnaire used in this study was developed specifically for the purposes of this research. The Supplementary Materials include the full study questionnaire and a comprehensive list of substances about which participants were questioned. (See Appendix Codebook)
