# Supplementary material for: Identifying subgroups of Chinese men who have sex with men based on sexual behavior and drug use patterns using a clustering analysis approach
Source: BMC Public Health. 2025 Apr 10;25:1353. doi: 10.1186/s12889-025-22388-x (PMC11983739; doi:10.1186/s12889-025-22388-x)
Supplement: Supplementary file 1 — Supplementary Material 1 [file 12889_2025_22388_MOESM1_ESM.docx]

**2022 Chinese MSM cross-sectional survey codebook (Date: 27May2022)**

|  | Variable | Code |
| --- | --- | --- |
|  | ID | Number |
| **A** | **Inclusion criteria** |  |
| A1 | How would you describe your assigned sex at birth? | 1=Male |
| A2 | In your lifetime, have you ever had anal sex with another man? | 1=Yes |
| A3 | Please enter your age: | Number  70=aged ≥ 70 yrs |
| **B** | **Sociodemographic characteristics** |  |
| B1 | What is your current legal marital status (referring to women)? | 1=Single  2=Engaged or Married  3=Separated or Divorced or Widowed |
| B2 | What is the highest level of education that you have completed? | 1=High school or below (including Zhongzhuan)  2=College/Bachelors (including Dazhuan)  3=Masters or above |
| B3 | What is your total individual monthly income from all sources? (RMB) | 1=<1500 RMB  2=1500-3000  3=3001-5000  4=5001-8000  5=>8001 |
| B4 | What is your gender identity? | 1=Male  2=Female  3=Transgender  4=Unsure/Other |
| B5 | What is your sexual orientation? | 1=Gay or Homosexual  2=Bisexual  3=Heterosexual  4=Unsure/Other |
| B6 | Have you ever told anyone about your sexuality or sexual history with men (except your sexual partner)? | 1=Yes  2=No (Skipped to C1) |
| B7 | Have you ever told health-care providers about your sexuality or sexual history with men? | 1=Yes  2=No  -3=Skipped |
| **C** | **Sexual intercourse history** |  |
| C1 | How old were you when you had sex with other person for the first time (including oral sex)? | Number |
| C2 | What is the assigned sex at birth of your first-time sexual partner? | 1=Male  2=Female |
| C3 | In the last six months, when you had anal sex with another man, what role did you assume? | 1=Mostly insertive (mostly 1)  2=Mostly receptive (mostly 0)  3=Both insertive and receptive in similar amounts (Both 1 and 0 in similar amounts) |
| C4 | In the last six months, how many male stable partners do you have in total? (Stable partners are sexual partners with stable relationships, including boyfriends with whom establish romantic relationships and other male sexual partners who maintain stable relationships.) | Number  One respondent self-reported in text.  Input “0” skipped to C6 |
| C5 | In the last six months, when you had anal sex with your male stable partner(s), how frequently did you use condoms? | 1=Never  2=Occasionally  3=Often  4=Always  -3=Skipped |
| C6 | In the last six months, how many male casual partners do you have in total? (Casual partners are who have been in a sexual relationship with you for less than or equal to 3 months, this also includes non-stable partners and male sex workers. | Number  One respondent self-reported in text.  Input “0” skipped to C8 |
| C7 | In the last six months, when you had anal sex with your male casual partner(s), how frequently did you use condoms? | 1=Never  2=Occasionally  3=Often  4=Always  -3=Skipped |
| C8 | What are the reasons that you did not use condoms when having anal sex? Please select all that apply. |  |
| C8_1 | *I use condoms every time* | 0=No  1=Yes  For detailed Chinese text of the option “Others, please specify:_____”, please contact SESH research assistants. |
| C8_2 | *Alcohol or drug effects* |  |
| C8_3 | *Sexual partner’s requirement* |  |
| C8_4 | *I trusted my partner(s) and feel myself will not get HIV* |  |
| C8_5 | *I perceived an increase in pleasure with unprotected intercourse* |  |
| C8_6 | *I had no condoms* |  |
| C8_7 | *I used PrEP* |  |
| C8_8 | *Others, please specify:____* |  |
| C9 | In general, where did you mainly have sex with your male sexual partners? Please select all that apply. |  |
| C9_1 | Home | 0=No  1=Yes  For detailed Chinese text of the option “Others, please specify:_____”, please contact SESH research assistants. |
| C9_2 | *Hotel* |  |
| C9_3 | *Bathhouse* |  |
| C9_4 | *Outdoor sex* |  |
| C9_5 | *Others, please specify:____* |  |
| C10 | In the last 6 months, have you received money, gifts, or favors in exchange for sex? | 1=Yes  2=No (Skipped to C12) |
| C11 | Is being paid money in exchange for sex your primary source of income? | 1=Yes  2=No  -3=Skipped |
| C12 | In the last 6 months, how often did you have group sex involving at least 2 people other than yourself at the same time? | 1=Never  2=Occasionally  3=Often  4=Always |
| C13 | In the last 6 months, have you had any vaginal sex? | 1=Yes  2=No (Skipped to D1) |
| C14 | In the last 6 months, how often did you use condoms when you had vaginal sex? | 1=Never  2=Occasionally  3=Often  4=Always  -3=Skipped |
| **D** | **Chemsex** |  |
| D1 | Have you **ever used** any of drugs or substances during sex? | 1=Yes  2=No (Skipped to D6) |
| D2 | Have you **ever used** any of the following drugs or substances during sex? Please select all that apply: |  |
| D2_1 | Alcohol | 0=No  1=Yes  -3=Skipped  For detailed Chinese text of the option “Others, please specify:_____”, please contact SESH research assistants. |
| D2_2 | *Poppers* |  |
| D2_3 | *Heroin* |  |
| D2_4 | *Crystal Meth (‘Ice’/’Cream’)* |  |
| D2_5 | *Marijuana* |  |
| D2_6 | *Ketamine* |  |
| D2_7 | *Ecstasy* |  |
| D2_8 | *GHB / GBL* |  |
| D2_9 | *Erectile dysfunction medication – e.g. Viagra* |  |
| D2_10 | *Others, please specify:* |  |
| D3 | Have you used any of the following drugs or substances during sex in the last 12 months? Please select all that apply: |  |
| D3_1 | *Alcohol* | 0=No  1=Yes  -3=Skipped  For detailed Chinese text of the option “Others, please specify:_____”, please contact SESH research assistants. |
| D3_2 | *Poppers* |  |
| D3_3 | *Heroin* |  |
| D3_4 | *Crystal Meth (‘Ice’/’Cream’)* |  |
| D3_5 | *Marijuana* |  |
| D3_6 | Ketamine |  |
| D3_7 | *Ecstasy* |  |
| D3_8 | *GHB / GBL* |  |
| D3_9 | *Erectile dysfunction medication – e.g. Viagra* |  |
| D3_10 | *Others, please specify:* |  |
| D3_11 | *I have not used any of the drugs mentioned above (Skipped to D5)* |  |
| D4 | In the last 12 months, how often did you use the following substances during sex, or for the purpose of sex? |  |
| D4_1 | Alcohol | 1=Never  2=Always  3=More than half the time  4=About half the time  5=Less than half the time  -3=Skipped |
| D4_2 | *Poppers* |  |
| D4_3 | *Heroin* |  |
| D4_4 | *Marijuana* |  |
| D4_5 | *Ketamine* |  |
| D4_6 | *Ecstasy* |  |
| D4_7 | *GHB / GBL* |  |
| D4_8 | *Erectile dysfunction medication – e.g. Viagra* |  |
| D4_9 | *Others (specified in D3)* |  |
| D5 | Have you ever injected any of substances? | 1=Yes  2=No (Skipped to E6)  -3=Skipped |
| D6 | Have you ever injected any of the following substances? Please select all that apply: |  |
| D6_1 | Alcohol | 0=No  1=Yes  -3=Skipped  For detailed Chinese text of the option “Others, please specify:_____”, please contact SESH research assistants. |
| D6_2 | *Poppers* |  |
| D6_3 | *Heroin* |  |
| D6_4 | *Crystal Meth (‘Ice’/’Cream’)* |  |
| D6_5 | *Marijuana* |  |
| D6_6 | *Ketamine* |  |
| D6_7 | *Ecstasy* |  |
| D6_8 | *GHB / GBL* |  |
| D6_9 | *Erectile dysfunction medication – e.g. Viagra* |  |
| D6_10 | *Please specify others: _______________* |  |
| D7 | Have you injected any of the above substances in the last 12 months? Please select all that apply: |  |
| D7_1 | *Alcohol* | 0=No  1=Yes  -3=Skipped  For detailed Chinese text of the option “Others, please specify:_____”, please contact SESH research assistants. |
| D7_2 | *Poppers* |  |
| D7_3 | *Heroin* |  |
| D7_4 | *Crystal Meth (‘Ice’/’Cream’)* |  |
| D7_5 | *Marijuana* |  |
| D7_6 | *Ketamine* |  |
| D7_7 | *Ecstasy* |  |
| D7_8 | *GHB / GBL* |  |
| D7_9 | *Erectile dysfunction medication – e.g. Viagra* |  |
| D7_10 | *Please specify others: _______________* |  |
| D7_11 | *I have not injected any of the substances mentioned above in the last 12 months （Skipped to E1）* |  |
| D8 | How often did you use more than one type of substance at the same time or simultaneously (i.e. mixing drugs or a ‘cocktail’ of drugs) during sex in the last 12 months? | 1=Always  2=More than half the time  3=Half the time  4=Less than half the time  5=Never (Skipped to E1)  -3=Skipped |
| D9 | How often did these chemsex sessions include 3 persons or more (i.e. orgies or group sex) in the last 12 months? | 1=Always  2=More than half the time  3=Half the time  4=Less than half the time  5=Never  -3=Skipped |
| **E** | **Most recent sexual activity with man** |  |
| E1 | Please recall the last time you had sex with a man. What was the relationship between that man and you? | 1=Stable partner  2=Friends with benefits  3=Casual partner  4=Commercial sex partners |
| E2 | Please recall the time when your most recent sex activity with a man. (If you did not have the specified sex or refused to answer, please fill in "-1"; filling in "0" means to having the sex on the day the questionnaire was completed) |  |
| E2_1 | *The last time you had sex with a man (including only French kiss) was ___ days ago.* | *Number (If you did not have the specified sex or refused to answer, please fill in "-1"; filling in "0" means to having the sex on the day the questionnaire was completed)* |
| E2_2 | *The last time I had the French kiss with a man was ___days ago.* |  |
| E2_3 | *The last time I gave oral sex to a man was ___ days ago.* |  |
| E2_4 | *The last time I was given oral sex to a man was __days ago* |  |
| E2_5 | *The last time I licked a man's anus was__ days ago* |  |
| E2_6 | *The last time my anus was licked by a man was__ days ago* |  |
| E2_7 | The last time I had anal sex with a man was__ days ago (my role was insertive) |  |
| E2_8 | *The last time I was given anal sex by a man was__ days ago (my role was receptive)* |  |
| E2_9 | *The last time I masturbated myself was__ days ago* |  |
| E2_10 | *The last time I masturbated for a man was ___ days ago* |  |
| E2_11 | *The last time I was masturbated by a man was ___ days ago* |  |
| E3 | Did you use drugs during last time you had sex with a man? | 1=Yes  2=No (Skipped to E6) |
| E4 | Which drugs did you use? (Please select all) |  |
| E4_1 | *Rush* | 0=No  1=Yes  -3=Skipped |
| E4_2 | *Crystal Meth (‘Ice’/’Cream’)* |  |
| E4_3 | *Ketamine* |  |
| E4_4 | *Ecstasy* |  |
| E4_5 | *Ya ba pills* |  |
| E4_6 | *Mixing drugs* |  |
| E4_7 | *DMT* |  |
| E4_8 | *GHB* |  |
| E4_9 | *Triazolam* |  |
| E4_10 | *Monkey dust* |  |
| E4_11 | *Others* |  |
| E5 | When did you use? | 1=Before sex  2=During sex  3=After sex  -3=Skipped |
| E6 | Please recall your most recent sexual activity, and then fill in the order in which your sexual activity occurred. |  |
| E6_1 | *Step 1* | 1=Kissed  2= masturbated myself  3= masturbated for a man  4= masturbated by a man  5= I was given oral sex  6= I gave oral sex  7= my anus was licked by a man  8= I licked a man’s anus  9= I was given anal sex by a man (my role was receptive)  10=I had anal sex with a man (my role was insertive)  11= Sex end (Skipped to F1)  -3=Skipped |
| E6_2 | *Step 2* |  |
| E6_3 | *Step 3* |  |
| E6_4 | *Step 4* |  |
| E6_5 | *Step 5* |  |
| E6_6 | *Step 6* |  |
| E6_7 | *Step 7* |  |
| E6_8 | *Step 8* |  |
| E6_9 | *Step 9* |  |
| E6_10 | *Step 10* |  |
| E6_11 | *Step 11* |  |
| E6_12 | *Step* ***12*** |  |
| **F** | **Internet usage** |  |
| F1 | How likely do you agree or disagree with the following statements? |  |
| F1_1 | *I use the Internet to get general health information.* | 1= Strong disagree  2= Disagree  3= Neutral  4= Agree  5= Strongly agree |
| F1_2 | *I use the Internet to get information on medicines/drugs.* |  |
| F1_3 | *I use the Internet to be equipped with information before/after doctor’s appointment.* |  |
| F1_4 | *I use the Internet to get descriptions of various diseases.* |  |
| F1_5 | *I use the Internet to get information on treatments/therapy/diagnosis.* |  |
| F1_6 | *I use the Internet to get information on how to care for oneself.* |  |
| F1_7 | *I use the Internet to decide about how to treat an illness.* |  |
| F1_8 | *I use the Internet to decide about whether or not to visit a doctor.* |  |
| F1_9 | *I use the Internet to understand how to deal with an illness.* |  |
| F1_10 | *I use the Internet to get information on hospitals/clinics/other health care facilities.* |  |
| F1_11 | *I use the Internet to get information on health management (exercise, abstinence from drinking, smoking, diet, nutrition, stress, mental health, etc).* |  |
| F2 | How likely do you agree or disagree with the following statements? |  |
| F2_1 | *I use the Internet to get online medical consultation from medical professionals.* | 1= Strong disagree  2= Disagree  3= Neutral  4= Agree  5= Strongly agree |
| F2_2 | *I use the Internet to interact with people with similar health conditions.* |  |
| F3_3 | *I use the Internet to use mail to communicate with a doctor or a doctor’s office.* |  |
| F4_4 | *I use the Internet to share and exchange experiences about health and diseases.* |  |
| **G** | **Ability to cope with adversity** |  |
| G1 | For the following statements, please mark the response which you agree with. |  |
| G1_1 | *I am able to adapt to change.* | 1= Not true at all  2= Rarely true  3= Sometimes true  4= Often true  5= True nearly all the time |
| G1_2 | *I can deal with whatever comes.* |  |
| G1_3 | *I try to see humorous side of problems.* |  |
| G1_4 | *I think coping with stress can strengthen me.* |  |
| G1_5 | *I tend to bounce back after illness or hardship.* |  |
| G1_6 | *I can achieve goals despite obstacles.* |  |
| G1_7 | *I can stay focused under pressure.* |  |
| G1_8 | *I am not easily discouraged by failure.* |  |
| G1_9 | *I think of self as strong person.* |  |
| G1_10 | *I can handle unpleasant feelings.* |  |
| **H** | **STI testing (HIV & syphilis & gonorrhea)** |  |
| H1 | In the last 6 months, have you been diagnosed with any sexually transmitted infections (STIs), other than HIV? | 1=Yes  2=No (Skipped to H3) |
| H2 | What type of STI have you been diagnosed with? |  |
| H2_1 | Anogenital warts | 0=No  1=Yes  -3=Skipped  For detailed Chinese text of the option “Others, please specify:_____”, please contact SESH research assistants. |
| H2_2 | Syphilis |  |
| H2_3 | Gonorrhea |  |
| H2_4 | Genital herpes |  |
| H2_5 | Chlamydia |  |
| H2_6 | Others, please specify: ___ |  |
| H2_7 | I don’t know |  |
| H3 | Have any of your sexual partners in the past 6 months been infected with an STI? | 1=Yes  2=No  3=Not sure |
| H4 | How regularly do you test for STI? | 1=I never test for STI. (Skipped to H6)  2=About once a year  3=About once every 6 months  4=About once every 3 months  5=About once a month |
| H5 | What type of institution do you mainly choose for STI testing? Please select all that apply. |  |
| H5_1 | *Public general hospitals* | 0=No  1=Yes  -3=Skipped  For detailed Chinese text of the option “Others, please specify:_____”, please contact SESH research assistants. |
| H5_2 | *Public specialty hospitals* |  |
| H5_3 | *Private hospital* |  |
| H5_4 | Public community-based outpatient clinic |  |
| H5_5 | *Private outpatient clinic* |  |
| H5_6 | *Self-testing* |  |
| H5_7 | *Other, please specify* |  |
| H6 | What are the reasons that you did not conduct STI testing? Please select all that apply. |  |
| H6_1 | *Not necessary* | 0=No  1=Yes  For detailed Chinese text of the option “Others, please specify:_____”, please contact SESH research assistants. |
| H6_2 | *Cost of STI testing is too expensive* |  |
| H6_3 | *Worried about privacy issues* |  |
| H6_4 | *Worried about being discriminated* |  |
| H6_5 | *No time* |  |
| H6_6 | *Other, please specify ______* |  |
| H7 | When was the last time you tested for gonorrhea? | 1=Never tested for Gonorrhea (Skipped to H15)  2=More than 12 months ago  3=In the last 7-12 months  4=In the last 4-6 months  5=In the last 3 months |
| H8 | How regularly do you test for gonorrhea? | 1=I do not test regularly for gonorrhea (Skipped to H10)  2=About once a year  3=About once every 6 months  4=About once every 3 months  5=About once a month  -3=Skipped |
| H9 | At your typical test for gonorrhoea, do you recall which sites on your body you tested for gonorrhea? Please select all that apply. |  |
| H9_1 | *Urethral (Swab / Urine)* | 0=No  1=Yes  -3=Skipped |
| H9_2 | *Rectal/Anal (Swab)* |  |
| H9_3 | *Pharyngeal/Oral (Swab)* |  |
| H10 | At your last test for gonorrhoea, do you recall which sites on your body you tested for gonorrhea? Please select all that apply. |  |
| H10_1 | *Urethral (Swab / Urine)* | 0=No  1=Yes  -3=Skipped |
| H10_2 | *Rectal/Anal (Swab)* |  |
| H10_3 | *Pharyngeal/Oral (Swab)* |  |
| H11 | Which of these statements best reflect your decision to get tested for gonorrhea, at your last test? | 1=I was referred to get tested by a doctor due to signs of gonorrhea  2=I decided to test for gonorrhea as I knew I was displaying symptoms/signs of gonorrhea  3=I decided to get tested for gonorrhea as part of my usual, routine check-up  4=I decided to get tested for gonorrhea because it was free  5=I decided to get tested for gonorrhea as I was getting into a new relationship  6=I am unable to recall  7=Other reasons, please specified  -3=Skipped  For detailed Chinese text of the option “Others, please specify:_____”, please contact SESH research assistants. |
| H12 | Which of these statements best reflect your typical decisions to get tested for gonorrhea? | 1=I was referred to get tested by a doctor due to signs of gonorrhea  2=I decided to test for gonorrhea as I knew I was displaying symptoms/signs of gonorrhea  3=I decided to get tested for gonorrhea as part of my usual, routine check-up  4=I decided to get tested for gonorrhea because it was free  5=I decided to get tested for gonorrhea as I was getting into a new relationship  6=I am unable to recall  7=Other reasons, please specified  -3=Skipped  For detailed Chinese text of the option “Others, please specify:_____”, please contact SESH research assistants. |
| H13 | Did you receive any treatment following your last gonorrhea test? | 1=No, I tested negative for gonorrhea (Skipped to H15)  2=No, I tested positive but did not receive any treatment for it by my doctor (Skipped to H15)  3=Yes, I tested negative but was still given treatment by my doctor  4=Yes, I tested positive and was given treatment by my doctor  5=Yes, I tested positive and bought treatment elsewhere  6=I am unable to recall (Skipped to H15)  -3=Skipped |
| H14 | Following your treatment, did you have to revisit the doctor due to a persisting infection? | 1=Yes, I revisited my doctor or another healthcare professional as the infection did not go away  2=No, my infection went away following treatment  3=I am unable to recall  -3=Skipped |
| H15 | Do you regularly go to Voluntary Counselling and Testing (VCT)? | 1=Yes  2=No  3=I do not go to VCT regularly |
| H16 | In the last 6 months, have your sexual partners been infected with HIV? | 1=Yes  2=No  3=Not sure |
| H17 | Have you heard about testing together? (Testing Together, previously known as Couples HIV Testing and Counseling, is a public health strategy that occurs when two or more persons who are in or planning to be in a sexual relationship receive HIV testing services together (including their HIV test results.) | 1=Yes  2=No |
| H18 | Are you willing to conduct testing together with your male partners? | 1=Yes  2=No (Skipped to H22) |
| H19 | What are the reasons that you would conduct testing together with your male partner(s)? Please select all that apply. |  |
| H19_1 | *Being able to know your own health and that of male partner(s)* | 0=No  1=Yes  -3=Skipped |
| H19_2 | *Feel societal accepted as a gay person* |  |
| H19_3 | *Can discuss HIV and other health issues with male partner(s)* |  |
| H19_4 | *Increase trust with your male partner(s)* |  |
| H19_5 | *Gain support from your male partner(s)* |  |
| H19_6 | *Laying the foundation for maintaining future relationships with male partner(s)* |  |
| H20 | When would you consider testing together with a male partner? | 1=Before establishing a new relationship with a partner  2=Shortly after starting a new relationship with a partner  3=Longer after a stabilized relationship with a partner  -3=Skipped |
| H21 | Where would you and your partner like to conduct testing together? | 1=CDC  2=Gay-friendly community-based organizations  3=STI clinics  4=General hospitals  5=Testing services provided in gay-friendly gathering places (bars, bathhouses, etc.)  6=Blood donation sites  7=Other, please specify:______  -3=Skipped  For detailed Chinese text of the option “Others, please specify:_____”, please contact SESH research assistants. |
| H22 | What are your main concerns about not conducting testing together? |  |
| H22_1 | *I feel myself have a higher risk of HIV infection* | 0=No  1=Yes  -3=Skipped  For detailed Chinese text of the option “Others, please specify:_____”, please contact SESH research assistants. |
| H22_2 | *I worry about possible breakup of partner relationship* |  |
| H22_3 | *Other, please specify ______* |  |
| H23 | When was the last time you tested for HIV? | 1=Never tested for HIV (Skipped to I1)  2=More than 12 months ago  3=In the last 7-12 months  4=In the last 4-6 months  5=In the last 3 months |
| H24 | Where was the last time you tested for HIV (including HIV self-testing)? | 1=CDC  2=Hospital/STI clinic  3=Gay-friendly community-based organization  4=Blood donation sites  5=Purchased/Used HIV self-testing kits  6=Testing services provided in gay-friendly gathering places  -3=Skipped |
| H25 | Have you ever conducted HIV self-testing | 1=Yes  2=No (Skipped to H27)  -3=Skipped |
| H26 | Why was your last HIV self-testing conducted? | 1=Regular test  2=Had unprotected sex  3=Felt unwell (cold, fever, etc.)  4=Wanted to know each other's HIV status before having sex with casual partners  5=Wanted to know each other’s HIV status with stable partners  6=Others, please specify:___  -3=Skipped  For detailed Chinese text of the option “Others, please specify:_____”, please contact SESH research assistants. |
| H27 | What was your last HIV testing result? | 1=Negative  2=Positive (Skipped to J1)  3=Prefer not to say  -3=Skipped |
| **I** | **PrEP** |  |
| **I1** | Have you heard of the following types of PrEP? |  |
| I1_1 | Taking one oral pill every day (“daily PrEP”) | 1= Yes  2=No  3=I am not sure  -3=Skipped |
| I1_2 | Taking oral pills around the time of sexual encounters (“on-demand PrEP”, “event-driven PrEP”, “2-1-1”) |  |
| I1_3 | Having injections every 2 months (“long-acting injectable PrEP”, “Cabotegravir”) |  |
| I2 | Do you know where and how to get oral PrEP currently? | 1= Yes  2=No  -3=Skipped |
| I3 | Have you spoken to a doctor or healthcare worker about starting PrEP? | 1= Yes  2=No  -3=Skipped |
| I4 | Have you ever taken PrEP? | 1= Yes (Skipped to I7)  2=No  -3=Skipped |
| I5 | Would you like to take PrEP but have not? | 1= Yes  2=No (Skipped to J1)  -3=Skipped |
| I6 | Why haven’t you taken PrEP? Please select all. |  |
| I6_1 | PrEP is not available where I live (Skipped to J1) | 1= Yes (Skipped to J1)  0=No (Skipped to J1)  -3=Skipped  For detailed Chinese text of the option “please write in how you take it____”, please contact SESH research assistants. |
| I6_2 | I don’t know where or how to get it (Skipped to J1) |  |
| I6_3 | It is too expensive (Skipped to J1) |  |
| I6_4 | I have not been able to get a prescription (Skipped to J1) |  |
| I6_5 | I’m worried about side effects (Skipped to J1) |  |
| I6_6 | I’m concerned about what my friends and family would think of me (Skipped to J1) |  |
| I6_7 | I don’t like taking pills on a regular basis (Skipped to J1) |  |
| I6_8 | I prefer to use condoms (Skipped to J1) |  |
| I6_9 | I don’t feel comfortable discussing my sexual risks with healthcare providers (Skipped to J1) |  |
| I6_10 | I was denied access to PrEP by a healthcare provider (Skipped to J1) |  |
| I6_11 | I am not at high risk of HIV (Skipped to J1) |  |
| I6_12 | Other (please write in):________ (Skipped to J1) |  |
| I7 | Are you currently taking PrEP? | 1=Yes, I am currently taking PrEP (including daily and on-demand PrEP)  2=No, I have stopped temporarily  3=No, I have stopped permanently  -3=Skipped |
| I8 | How do you currently take PrEP? | 1=I take it daily/most days (“daily PrEP”)  2=I take it around the time of sex (“on-demand PrEP”, “event-driven PrEP”, “2-1-1”)  3=I take it another way (please write in how you take it): _________  -3=Skipped  For detailed Chinese text of the option “please write in how you take it____”, please contact SESH research assistants. |
| I9 | Where do you usually get your PrEP pills? Please select all. |  |
| I9_1 | *As part of a research project* | 0=No  1=Yes  -3=Skipped  For detailed Chinese text of the option “please write in the other place(s) you got your PrEP pills ____”, please contact SESH research assistants. |
| I9_2 | *Directly from my doctor/clinic* |  |
| I9_3 | *Directly from a community-based organization* |  |
| I9_4 | *From my local pharmacy with a PrEP prescription* |  |
| I9_5 | *From my local pharmacy without a PrEP prescription* |  |
| I9_6 | *I bought it online with a PrEP prescription* |  |
| I9_7 | *I bought it online without a PrEP prescription* |  |
| I9_8 | *From a friend or sex partner* |  |
| I9_9 | *I used PEP pills as PrEP* |  |
| I9_10 | *Another country* |  |
| I9_11 | *Other, please write in the other place(s) you got your PrEP pills ( _____________)* |  |
| **J** | **Knowledge and attitude towards HIV** |  |
| **J1** | Please select the response which you agree with. |  |
| J1_1 | *A person infected with HIV can be seen by the appearance.* | 1=Agree  2=Disagree  3=Not sure |
| J1_2 | *Mosquito bites can transmit HIV.* |  |
| J1_3 | *You can get infected with HIV by eating with someone who is infected with HIV or has AIDS.* |  |
| J1_4 | *Flu-like symptoms are a classical sign of early stage HIV infection.* |  |
| J1_5 | *You can get AIDS if you receive blood products infected with HIV.* |  |
| J1_6 | *It is possible to get AIDS by sharing syringes and drugs with HIV-infected people.* |  |
| J1_7 | *Children born to HIV-infected women are at risk for AIDS.* |  |
| J1_8 | *Treatment with HIV drugs can make a person non-contagious to their sexual partners.* |  |
| J1_9 | *Washing or douching the anus before or after sex reduces risk of HIV infection* |  |
| J1_10 | *Having sex with only one partner can reduce the risk of HIV transmission.* |  |
| J1_11 | *Condomless anal sex has the same risk of HIV transmission as condomless vaginal sex.* |  |
| J1_12 | *Proper use of condoms can reduce the risk of HIV transmission.* |  |
| J2 | Do you agree with the following statement: “People with HIV are most contagious to their sexual partners immediately after they have become infected (acute period).”? | 1=Yes  2=No  3=Not sure |
| J3 | Which is the following affect a person’s risk of HIV? | 1= a.Their sexual network (their partners and partner’s partners)  2=b.Their access to healthcare  3=c.Their condom use behaviors  4=d.All of the above  5=e.A and C  6=f. B and C |
| J4 | If you think you may have put yourself at risk, you could be eligible for post-exposure prophylaxis (PEP) as long as it has been less than: | 1=24 hours  2=36 hours  3=72 hours  4=One week  5=I’m not sure |
| J5 | What is a “window period” when getting an HIV test? | 1=The time it takes to receive your test results  2=The time between HIV infection and when symptoms first appear  3=The time it takes from infection to when a test will produce accurate results  4=I’m not sure |
| **K** | **GBM community attachment scale & homophobia experience** |  |
| K1_1 | How much do you feel part of a gay/bisexual community? | 1=Not at all  2=A little bit  3=Somewhat  4=Quite a bit  5=Completely |
| K1_2 | How many of your friends are gay/bisexual? | 1=None of them  2=Some  3=About half of them  4=Almost all  5=All of them |
| K1_3 | How much of your free time do you spend with gay or bisexual men? | 1=None of it  2=Some  3=About half of it  4=Almost all  5=All of it |
| K1_4 | How much of your time do you spend giving back to the gay/bisexual community? (e.g. volunteering, advocacy, passing on health information to others) | 1=None of it  2=Some  3=About half of it  4=Almost all  5=All of it |
| K2 | Have you ever been discriminated against because of your sexual orientation? Sexual orientation refers to a person’s physical, romantic, and/or emotional attraction towards other people. | 1=Yes  2=No (Skipped to K5) |
| K3 | For the following 9 statements, please mark whether you have experienced any of the following on the grounds of your sexual orientation during adolescence (aged between 10-19 years old)? |  |
| K3_1 | *I have been insulted or threatened* | 1=Yes  2=No  3=Unsure  -3=Skipped |
| K3_2 | *I have been beaten, pushed or kicked* |  |
| K3_3 | *My belongings have been destroyed or damaged* |  |
| K3_4 | *I was not given an opportunity for school or was dismissed from my school.* |  |
| K3_5 | *I was treated in a discriminatory way by a healthcare professional* |  |
| K3_6 | *I was denied medical treatment* |  |
| K3_7 | *I was jailed, prosecuted or denied legal services* |  |
| K3_8 | *I was asked to leave my home or thrown out of my accommodations* |  |
| K3_9 | *I was forced to engage in a sexual act, sexually assaulted, or raped* |  |
| K4 | When was the last time you were discriminated against because of your sexual orientation? | 1=In the last year  2=More than 1 year ago  3=Don’t know  4=Prefer not to answer  -3=Skipped |
| K5 | Have you ever been discriminated against because of your gender identity? Gender identity refers to someone’s individual and internal experience of gender, which may or may not correspond with their sex assigned at birth. | 1=Yes  2=No (Skipped to L1) |
| K6 | For the following 9 statements, please mark whether you have experienced any of the following on the grounds of your gender identity during adolescence (aged between 10-19 years old)? |  |
| K6_1 | *I have been insulted or threatened* | 1=Yes  2=No  3=Unsure  -3=Skipped |
| K6_2 | *I have been beaten, pushed or kicked* |  |
| K6_3 | *My belongings have been destroyed or damaged* |  |
| K6_4 | *I was not given an opportunity for school or was dismissed from my school.* |  |
| K6_5 | *I was treated in a discriminatory way by a healthcare professional* |  |
| K6_6 | *I was denied medical treatment* |  |
| K6_7 | *I was jailed, prosecuted or denied legal services* |  |
| K6_8 | *I was asked to leave my home or thrown out of my accommodations* |  |
| K6_9 | *I was forced to engage in a sexual act, sexually assaulted, or raped* |  |
| K7 | When was the last time you were discriminated against because of your gender identity? | 1=In the last year  2=More than 1 year ago  3=Don’t know  4=Prefer not to answer  -3=Skipped |
| K8 | For the following 12 statements, please mark indicate how you feel about each statement. |  |
| K8_1 | *There is a special person who is around when I*  *am in need.* | 1=Very Strongly Disagree  2=Strongly Disagree  3=Mildly Disagree  4=Neutral  5=Mildly Agree  6=Strongly Agree  7=Very Strongly Agree  -3=Skipped |
| K8_2 | *There is a special person with whom I can share*  *my joys and sorrows.* |  |
| K8_3 | *My family really tries to help me.* |  |
| K8_4 | *I get the emotional help and support I need from*  *my family.* |  |
| K8_5 | *I have a special person who is a real source of*  *comfort to me.* |  |
| K8_6 | *My friends really try to help me.* |  |
| K8_7 | *I can count on my friends when things go wrong.* |  |
| K8_8 | *I can talk about my problems with my family.* |  |
| K8_9 | *I have friends with whom I can share my joys*  *and sorrows.* |  |
| K8_10 | *There is a special person in my life who cares*  *about my feelings.* |  |
| K8_11 | *My family is willing to help me make decisions.* |  |
| K8_12 | *I can talk about my problems with my friends.* |  |
| **L** | **Chronic disease history** |  |
| L1_1 | Height _____ cm | Number |
| L1_2 | Weight ____kg | Number |
| L2 | Do you have the following chronic disease history or health status history? |  |
| L2_1 | *Hypertension* | 1=Yes  2=No |
| L2_2 | *Diabetes* |  |
| L2_3 | *Thyroid conditions (excluding cancer)* |  |
| L2_4 | *Asthma* |  |
| L2_5 | *Other chronic respiratory disease cause breathing symptoms* |  |
| L2_6 | *Cancer* |  |
| L2_7 | *Liver disease (with affected liver function)* |  |
| L2_8 | *Haematological disease* |  |
| L2_9 | *Heart disease* |  |
| L2_10 | *Kidney disease* |  |
| L2_11 | *Neurological problems affecting speech, swallow, mobility, breathing* |  |
| L2_12 | *Rheumatoid* |  |
| L2_13 | *Lupus* |  |
| L2_14 | *Psoriasis* |  |
| L2_15 | *Immunosuppressive conditions or drugs* |  |
| L2_16 | *Spleen diseases (excluding traumatic splenectomy)* |  |
| L2_17 | *Organ transplant* |  |
| L2_18 | *Do you smoke?* |  |
| L3 | In your daily life, do you regularly check your blood pressure? | 1=Yes  2=No |
| L4 | In your daily life, do you regularly check your blood glucose? | 1=Yes  2=No |
| L5 | Do you know that MSM is a high-risk group for HIV/AIDS infection? | 1=Yes  2=No |
| L6 | Do you know that for people living with HIV, the risk of other chronic diseases (including cancer, diabetes, cardiovascular diseases, etc.) will increase correspondingly due to the invasion of HIV and the damage of the immune system? | 1=Yes  2=No |
| L7 | Do you know that for people living with HIV who are on ART, due to the antiretroviral treatments, the risk of other chronic diseases (including hyperlipidemia and diabetes) will increase? | 1=Yes  2=No |
| **M** | **Evaluation of local CDC or gay-led organization’s STI education and health promotion work** |  |
| M1_1 | Your current residence: Province _________ | 1=Anhui  2=Beijing  3=Chongqing  4=Fujian  5=Gansu  6=Guangdong  7=Guangxi  8=Hainan  9=Hebei  10=Heilongjiang  11=Henan  12=Hubei  13=Hunan  14=Jiangsu  15=Jiangxi  16=Inner Mongolia  17=Qinghai  18=Shandong  19=Jilin  20=Liaoning  21=Shanghai  22=Shanxi  23=Shaanxi  24=Sichuan  25=Tianjin  26=Xinjiang  27=Tibet  28=Yunnan  29=Zhejiang  30=Oversea |
| M1_2 | Your current residence: City _________ | (For more information in Chinese, please contact the research assistants) |
| M2 | Have you ever received the health publicity materials, health education such as information about STI (e.g., HIV/syphilis) from your local CDC or gay-led organization? This includes but not limited to: brochures, WeChat articles, consultation services on STI-related issues, community health popularization activities, STI education lectures, health promotion e-mail/messages, etc.; whether you receive them actively or passively. | 1=Yes  2=No (Skipped to M4) |
| M3 | Please evaluate on the health publicity, STI education work from your local CDC or gay-led organization. |  |
| M3_1 | The frequency of these health promotion and education science is high. | 1=Strongly disagree  2=disagree  3=neutral  4=agree  5=Strongly agree  -3=Skipped |
| M3_2 | The coverage of these health promotion and education science is high among local MSM. |  |
| M3_3 | These health promotion and education science are effective. |  |
| M3_4 | I am satisfied with these health promotion and education science. |  |
| M4 | Have you ever participated in the health promotion and intervention programs, STI prevention programs offered by your local CDC or gay-led organization? This includes but not limited to: STI testing, self-testing, secondary distribution of self-testing, PrEP, partner notification, etc.; whether you actively signed up to participate or received invitation to these programs. | 1=Yes  2=No (Skipped to N1) |
| M5 | Please evaluate on the health promotion and intervention programs, STI prevention programs offered by your local CDC or gay-led organization. |  |
| M5_1 | The frequency of these programs is high. | 1=Strongly disagree  2=disagree  3=neutral  4=agree  5=Strongly agree  -3=Skipped |
| M5_2 | The coverage of these programs is high among local MSM. |  |
| M5_3 | These programs are effective. |  |
| M5_4 | I am satisfied with these programs. |  |
| **N** | **Antibiotic and mouthwash use** |  |
| N1 | Have you used any antibiotics in the past three months? | 1=Yes  2=No (Skipped to N3) |
| N2_1 | Name of the antibiotic you used | Character  -3=Skipped  (For more information in Chinese, please contact the research assistants)  -3=Skipped  (For more information in Chinese, please contact the research assistants)  -3=Skipped  (For more information in Chinese, please contact the research assistants) |
| N2_2 | Date of use |  |
| N2_3 | Reasons for use |  |
| N3 | How often have you used mouthwash in the past three months? | 1= At least twice a day  2=Once a day  3= Several times per week  4=Once a week  5= Several times in the last three months  6= Never used in the last three months (Skipped to O1) |
| N4 | Please fill in the brand of the mouthwash you usually use (you can fill in multiple branches) | Character  -3=Skipped  (For more information in Chinese, please contact the research assistants) |
| N5 | In which of the following situations do you use mouthwash? (You can choose more than one) |  |
| N5_1 | *Before having sex* | 0=No  1=Yes  -3=Skipped |
| N5_2 | *After having sex* |  |
| N5_3 | *Not associated with sexual intercourse, used in the morning and evening when washing up/washing up after meals* |  |
| **O** | **Feasibility of COVID-19 self-testing kits** |  |
| O1 | Have you heard of the COVID-19 antigen self-testing kits? | 1=Yes  2=No  -3=Skipped |
| O2 | Are you currently informed about the channels and ways to obtain the COVID-19 antigen self-testing kits? | 1=Yes  2=No  -3=Skipped |
| O3 | Would you use the COVID-19 antigen self-testing kits? | 1=Yes  2=No  3=Not sure (skipped to O5)  -3=Skipped |
| O4 | What are your reasons for not using the COVID-19 antigen self-testing kits? |  |
| O4_1 | *It is difficult for me to read the self-test kit instructions* | 0=No  1=Yes  -3=Skipped  (For more information about O4_8 in Chinese, please contact the research assistants) |
| O4_2 | *I am afraid to swab my nasal area (note: the currently listed self-test kits use a nasal swab)* |  |
| O4_3 | *I think the self-test kits is too expensive (Note: the current pricing of the listed single test kit ranges from RMB17 to 32.50)* |  |
| O4_4 | *I think it takes too long for me to wait from purchasing the kit online to receiving it* |  |
| O4_5 | *I feel suspicious about the accuracy of the kits (Note: The self-test kits listed in China comply with the recommendations of the World Health Organization: the sensitivity of the test is not less than 80% and the specificity is not less than 97%.)* |  |
| O4_6 | *I don't think using the self-test kits will reduce the number of times I have to visit the hospital for COVID testing* |  |
| O4_7 | *I don't think I'm going to get infected, and therefore there is no need for testing* |  |
| O4_8 | *Others, please specify:* |  |
| O5 | What are your reasons for using the COVID-19 antigen self-testing kits? |  |
| O5_1 | *It is easy for me to read the self-test kit instructions* | 0=No  1=Yes  -3=Skipped  (For more information about O5_8 in Chinese, please contact the research assistants) |
| O5_2 | *It is simple for me to use the self-test kit and it is convenient to use for different scenarios* |  |
| O5_3 | *I think I am at risk of infection and the self-test kit will meet my daily testing needs* |  |
| O5_4 | *I think I can afford the self-test kit (note: the current pricing of the listed single test kit ranges from RMB25 to 32.50)* |  |
| O5_5 | *I think using self-test kits saves time cost of waiting for results (Note: 15-20 minutes for results with proper use of self-test kits)* |  |
| O5_6 | *Self-test kits can be purchased through retail pharmacies, online platforms, and other channels, reducing the number of times I have to visit hospitals for testing and transportation costs* |  |
| O5_7 | *I feel confident about the accuracy of the kits (Note: The self-test kits listed in China comply with the recommendations of the World Health Organization: the sensitivity of the test is not less than 80% and the specificity is not less than 97%.)* |  |
| O5_8 | *Others, please specify:* |  |
| O6 | Do you support the promotion of the COVID-19 antigen self-test kits? | 1=Yes  2=No  -3=Skipped |
| **P** | **Mobility** |  |
| P1 | [multiple choices & fill in blanks] In the recent 12 months, which of the following provinces/municipalities/provincial districts have your gay friends from other provinces visited your province and met with you (including but not limited to meeting, chatting, dining out, having sex, hanging out, etc.)? How many times did you meet each of them?  (For example, in the past 12 months, Qiang and Hua, your gay friends from Guangdong Province, have visited you once together, and then Qiang has visited you twice alone, so the total number of visits in Guangdong Province is 4. Please leave the option for your own province blank.) |  |
| P1_1 | Anhui | 0=No  1=Yes  (For more information about total number of visits, please contact the research assistants) |
| P1_2 | Beijing |  |
| P1_3 | Chongqing |  |
| P1_4 | Fujian |  |
| P1_5 | Gansu |  |
| P1_6 | Guangdong |  |
| P1_7 | Guangxi |  |
| P1_8 | Guizhou |  |
| P1_9 | Hainan |  |
| P1_10 | Hebei |  |
| P1_11 | Heilongjiang |  |
| P1_12 | Henan |  |
| P1_13 | Hongkong |  |
| P1_14 | Hubei |  |
| P1_15 | Hunan |  |
| P1_16 | Jiangsu |  |
| P1_17 | Jiangxi |  |
| P1_18 | Jilin |  |
| P1_19 | Liaoning |  |
| P1_20 | Macau |  |
| P1_21 | Inner Mongolia |  |
| P1_22 | Ningxia |  |
| P1_23 | Qinghai |  |
| P1_24 | Shandong |  |
| P1_25 | Shanghai |  |
| P1_26 | Shanxi |  |
| P1_27 | Shaanxi |  |
| P1_28 | Sichuan |  |
| P1_29 | Taiwan |  |
| P1_30 | Tianjin |  |
| P1_31 | Xinjiang |  |
| P1_32 | Tibet |  |
| P1_33 | Yunnan |  |
| P1_34 | Zhejiang |  |
| P1_35 | No visitor from other provinces |  |
| P2 | [multiple choices & fill in blanks] In the recent 12 months, which of the following provinces/municipalities/provincial districts have you traveled to and met with your local gay male friends (including, but not limited to, meeting, chatting，dining out, having sex, hanging out, etc.)? How many meetings were there?  (For example, if you have traveled to Sichuan Province twice in the past 12 months, the first time to meet your local gay male friend Zhang San, and the second time to meet Zhang San and Li Si, then the total number of visits to Sichuan Province is 3. Please leave the option for your own province blank.) |  |
| P2_1 | Anhui | 0=No  1=Yes  (For more information about total number of visits, please contact the research assistants) |
| P2_2 | Beijing |  |
| P2_3 | Chongqing |  |
| P2_4 | Fujian |  |
| P2_5 | Gansu |  |
| P2_6 | Guangdong |  |
| P2_7 | Guangxi |  |
| P2_8 | Guizhou |  |
| P2_9 | Hainan |  |
| P2_10 | Hebei |  |
| P2_11 | Heilongjiang |  |
| P2_12 | Henan |  |
| P2_13 | Hongkong |  |
| P2_14 | Hubei |  |
| P2_15 | Hunan |  |
| P2_16 | Jiangsu |  |
| P2_17 | Jiangxi |  |
| P2_18 | Jilin |  |
| P2_19 | Liaoning |  |
| P2_20 | Macau |  |
| P2_21 | Inner Mongolia |  |
| P2_22 | Ningxia |  |
| P2_23 | Qinghai |  |
| P2_24 | Shandong |  |
| P2_25 | Shanghai |  |
| P2_26 | Shanxi |  |
| P2_27 | Shaanxi |  |
| P2_28 | Sichuan |  |
| P2_29 | Taiwan |  |
| P2_30 | Tianjin |  |
| P2_31 | Xinjiang |  |
| P2_32 | Tibet |  |
| P2_33 | Yunnan |  |
| P2_34 | Zhejiang |  |
| P2_35 | No visitor from other provinces |  |
