# Supplementary material for: Identifying subgroups of Chinese men who have sex with men based on sexual behavior and drug use patterns using a clustering analysis approach
Source: BMC Public Health. 2025 Apr 10;25:1353. doi: 10.1186/s12889-025-22388-x (PMC11983739; doi:10.1186/s12889-025-22388-x)
Supplement: Supplementary file 2 — Supplementary Material 2 [file 12889_2025_22388_MOESM2_ESM.docx]

**­­­**
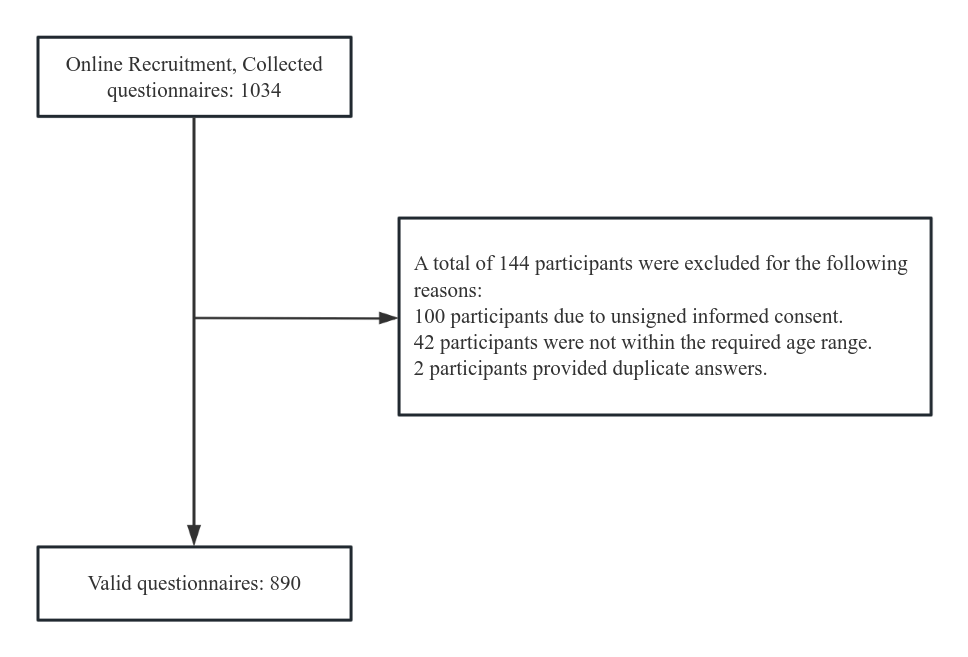


Figure S1：Study flowchart


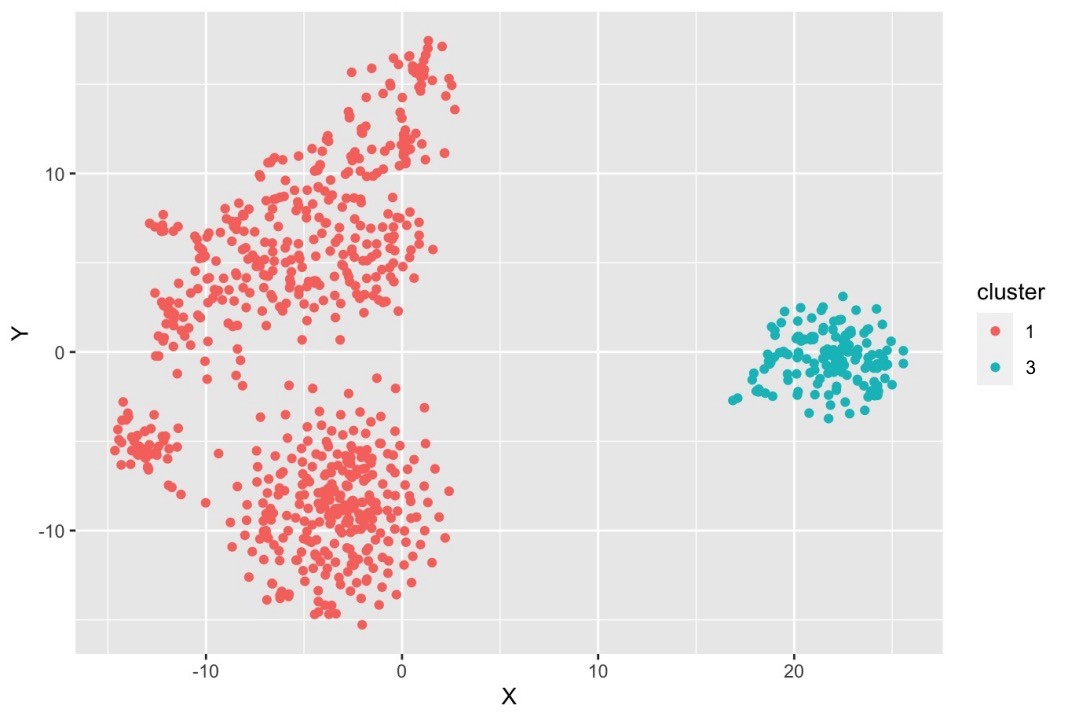


Figure S2: Reduced dimensional scatter plot of Gower clustering results


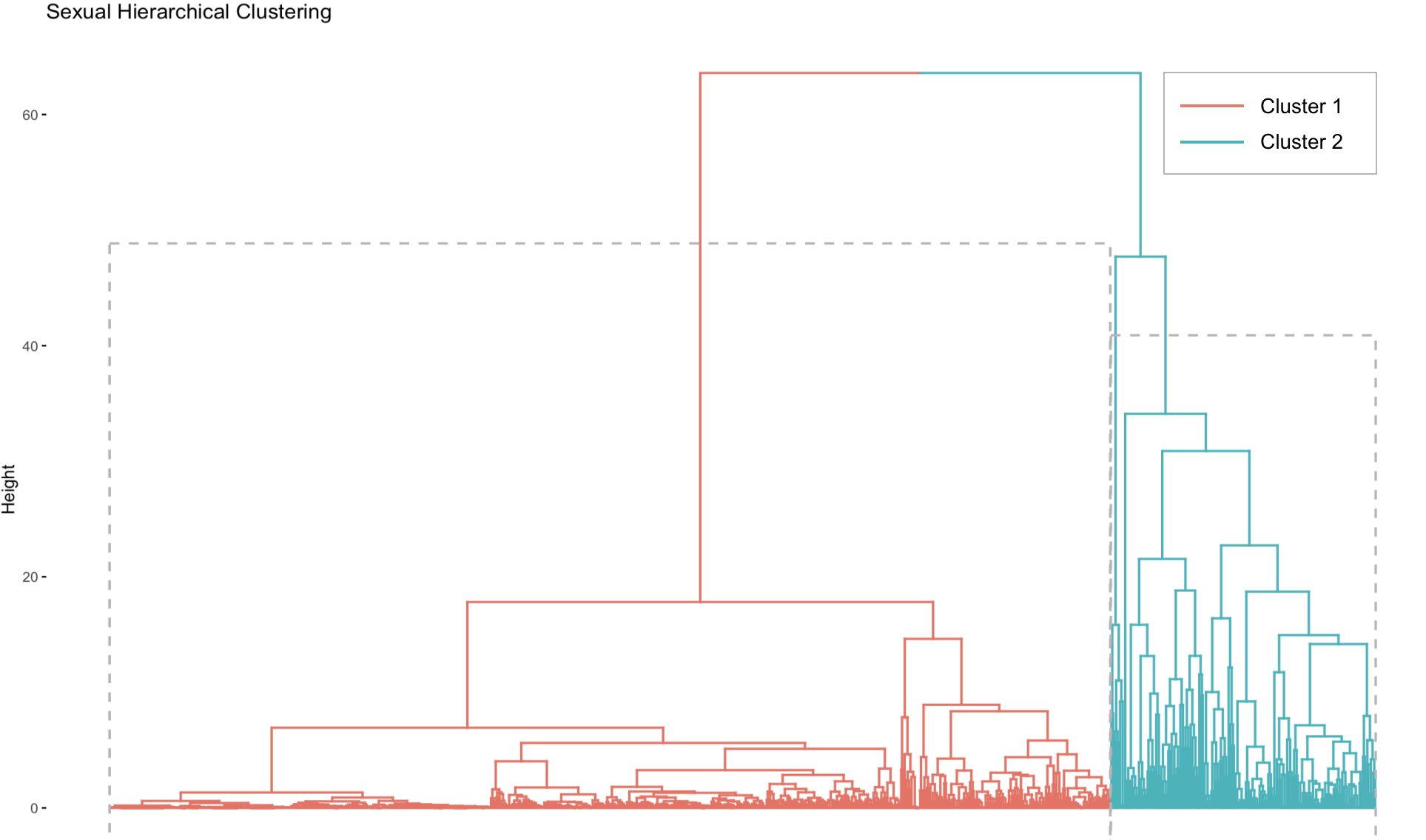


Figure S3: Reduced dimensional scatter plot of hierarchical clustering results

| Table S1: Frequency of Substance Use During Sexual Encounters in the Last 12 Months Across Clusters | | | | | |
| --- | --- | --- | --- | --- | --- |
| **Category** | Cluster 1 | Cluster 2 | Cluster 3 | Chi-Squared | P-value |
| In the last 12 months, how often did you use the following substances during sex |  |  |  |  |  |
| Poppers |  |  |  | 27.41 | 0.0022 |
| Skipped | 327(56.28%) | 90(58.44%) | 61(39.35%) |  |  |
| Never | 55(9.47%) | 17(11.04%) | 13(8.39%) |  |  |
| Always | 50(8.61%) | 13(8.44%) | 20(12.90%) |  |  |
| More than half the time | 28(4.82%) | 7(4.55%) | 20(12.90%) |  |  |
| About half the time | 39(6.71%) | 8(5.19%) | 14(9.03%) |  |  |
| Less than half the time | 82(14.11%) | 19(12.34%) | 27(17.42%) |  |  |
| Heroin |  |  |  | 21.14 | 0.0202 |
| Skipped | 327(56.28%) | 90(58.44%) | 61(39.35%) |  |  |
| Never | 246(42.34%) | 64(41.56%) | 94(60.65%) |  |  |
| Always | 0(0.00%) | 0(0.00%) | 0(0.00%) |  |  |
| More than half the time | 0(0.00%) | 0(0.00%) | 0(0.00%) |  |  |
| About half the time | 0(0.00%) | 0(0.00%) | 0(0.00%) |  |  |
| Less than half the time | 8(1.38%) | 0(0.00%) | 0(0.00%) |  |  |
| Marijuana |  |  |  | 21.38 | 0.0186 |
| Skipped | 327(56.28%) | 90(58.44%) | 61(39.35%) |  |  |
| Never | 245(42.17%) | 64(41.56%) | 94(60.65%) |  |  |
| Always | 0(0.00%) | 0(0.00%) | 0(0.00%) |  |  |
| More than half the time | 0(0.00%) | 0(0.00%) | 0(0.00%) |  |  |
| About half the time | 1(0.17%) | 0(0.00%) | 0(0.00%) |  |  |
| Less than half the time | 8(1.38%) | 0(0.00%) | 0(0.00%) |  |  |
| Ketamine |  |  |  | 21.83 | 0.016 |
| Skipped | 327(56.28%) | 90(58.44%) | 61(39.35%) |  |  |
| Never | 245(42.17%) | 64(41.56%) | 94(60.65%) |  |  |
| Always | 0(0.00%) | 0(0.00%) | 0(0.00%) |  |  |
| More than half the time | 0(0.00%) | 0(0.00%) | 0(0.00%) |  |  |
| About half the time | 0(0.00%) | 0(0.00%) | 0(0.00%) |  |  |
| Less than half the time | 9(1.55%) | 0(0.00%) | 0(0.00%) |  |  |
| Ecstasy |  |  |  | 21.83 | 0.016 |
| Skipped | 327(56.28%) | 90(58.44%) | 61(39.35%) |  |  |
| Never | 245(42.17%) | 64(41.56%) | 94(60.65%) |  |  |
| Always | 0(0.00%) | 0(0.00%) | 0(0.00%) |  |  |
| More than half the time | 0(0.00%) | 0(0.00%) | 0(0.00%) |  |  |
| About half the time | 0(0.00%) | 0(0.00%) | 0(0.00%) |  |  |
| Less than half the time | 9(1.55%) | 0(0.00%) | 0(0.00%) |  |  |
| GHB / GBL |  |  |  | 21.38 | 0.0186 |
| Skipped | 327(56.28%) | 90(58.44%) | 61(39.35%) |  |  |
| Never | 245(42.17%) | 64(41.56%) | 94(60.65%) |  |  |
| Always | 0(0.00%) | 0(0.00%) | 0(0.00%) |  |  |
| More than half the time | 0(0.00%) | 0(0.00%) | 0(0.00%) |  |  |
| About half the time | 1(0.17%) | 0(0.00%) | 0(0.00%) |  |  |
| Less than half the time | 8(1.38%) | 0(0.00%) | 0(0.00%) |  |  |
| Erectile dysfunction medication – e.g. Viagra |  |  |  | 20.99 | 0.0211 |
| Skipped | 327(56.28%) | 90(58.44%) | 61(39.35%) |  |  |
| Never | 158(27.19%) | 45(29.22%) | 65(41.94%) |  |  |
| Always | 6(1.03%) | 1(0.65%) | 2(1.29%) |  |  |
| More than half the time | 7(1.20%) | 3(1.95%) | 4(2.58%) |  |  |
| About half the time | 22(3.79%) | 5(3.25%) | 8(5.16%) |  |  |
| Less than half the time | 61(10.50%) | 10(6.49%) | 15(9.68%) |  |  |
| Others (specified in D3) |  |  |  | 28.4 | 0.0016 |
| Skipped | 327(56.28%) | 90(58.44%) | 61(39.35%) |  |  |
| Never | 235(40.45%) | 64(41.56%) | 92(59.35%) |  |  |
| Always | 1(0.17%) | 0(0.00%) | 0(0.00%) |  |  |
| More than half the time | 1(0.17%) | 0(0.00%) | 1(0.65%) |  |  |
| About half the time | 3(0.52%) | 0(0.00%) | 2(1.29%) |  |  |
| Less than half the time | 14(2.41%) | 0(0.00%) | 0(0.00%) |  |  |

Table S2: Estimated number of sexual acts in the past twelve months across three clusters

| **Category** | **Overall (Mean, 95% CI)** | **Cluster 1 (Mean, 95% CI)** | **Cluster 2 (Mean, 95% CI)** | **Cluster 3 (Mean, 95% CI)** | **H** | **P-value** |
| --- | --- | --- | --- | --- | --- | --- |
| any sex act | 52.53 (52.65, 88.61) | 56.10 (53.39, 73.33) | 21.22 (20.80, 35.35) | 61.30 (54.31, 91.13) | 77.97 | <0.0001 |
| kissing | 50.20 (52.26, 78.11) | 54.10 (52.96, 68.10) | 19.14 (18.31, 34.16) | 55.47 (52.25, 76.24) | 61.32 | <0.0001 |
| receptive oral sex | 46.97 (51.44, 59.77) | 48.41 (50.74, 54.00) | 15.67 (13.38, 32.94) | 54.87 (52.21, 70.29) | 60.71 | <0.0001 |
| insertive oral sex | 49.38 (52.07, 73.72) | 52.77 (52.33, 65.73) | 18.24 (16.86, 34.79) | 53.51 (51.66, 70.63) | 53.01 | <0.0001 |
| rimming | 34.90 (45.18, 25.03) | 31.03 (40.81, 23.48) | 4.37 (2.24, 31.77) | 35.80 (30.22, 42.53) | 85.16 | <0.0001 |
| be rimmed | 34.99 (46.39, 25.57) | 33.51 (42.53, 26.56) | 6.80 (3.72, 30.72) | 36.93 (32.12, 43.70) | 69.13 | <0.0001 |
| insertive anal sex | 43.24 (50.35, 41.00) | 44.35 (48.89, 43.55) | 6.72 (4.67, 25.47) | 46.04 (48.51, 48.67) | 92.59 | <0.0001 |
| receptive anal sex | 38.16 (48.23, 31.19) | 38.67 (45.60, 34.22) | 9.20 (6.04, 28.36) | 45.70 (46.69, 48.53) | 63.19 | <0.0001 |
| masturbation | 88.80 (153.18, 59.50) | 82.94 (119.90, 62.81) | 118.52 (70.32, 175.75) | 97.46 (147.79, 65.33) | 328.92 | <0.0001 |
| masturbation for | 45.58 (51.28, 43.76) | 45.90 (49.84, 45.29) | 16.84 (12.53, 33.38) | 45.90 (48.63, 46.69) | 21.27 | <0.0001 |
| masturbation by | 46.18 (51.53, 46.58) | 46.99 (50.15, 47.34) | 20.48 (15.04, 36.11) | 46.40 (49.20, 46.95) | 14.09 | <0.0001 |

Table S3: Median number of days since last sexual acts across three clusters

| **Category** | **Overall (Median, IQR)** | **Cluster 1 (Median, IQR)** | **Cluster 2 (Median, IQR)** | **Cluster 3 (Median, IQR)** | **H** | **P-value** |
| --- | --- | --- | --- | --- | --- | --- |
| any sex act | 14 (3, 60) | 10 (3, 30) | 56 (15, 100) | 7 (3, 30) | 48.79 | <0.0001 |
| kissing | 15 (3.25, 69.75) | 10 (3, 50) | 60 (20, 120) | 10 (3, 30) | 41.83 | <0.0001 |
| receptive oral sex | 22.5 (5, 167.25) | 15 (4, 365) | 60 (30, 180) | 9.5 (3, 41.75) | 38.55 | <0.0001 |
| insertive oral sex | 15 (4, 81.5) | 10 (3, 40) | 60 (20, 150) | 10 (3.5, 40.25) | 41.06 | <0.0001 |
| rimming | 365 (28.5, 365) | 365 (15, 365) | 365 (160.5, 365) | 100 (10, 365) | 12.63 | 0.002 |
| be rimmed | 200 (15, 365) | 365 (10, 365) | 300 (89.5, 365) | 60 (7, 365) | 22.28 | <0.0001 |
| insertive anal sex | 30 (7, 365) | 25 (5, 365) | 180 (60, 365) | 25 (5, 190) | 39.06 | <0.0001 |
| receptive anal sex | 60 (10, 365) | 35 (7, 365) | 167 (60, 365) | 30 (5, 365) | 27.96 | <0.0001 |
| masturbation | 3 (1, 7) | 3 (1, 7) | 2 (1, 4) | 3 (1, 7) | 7.23 | 0.027 |
| masturbation for | 30 (6, 365) | 30 (4, 365) | 60 (30, 200) | 22.5 (5, 365) | 5.88 | 0.052 |
| masturbation by | 30 (5, 365) | 26 (4, 365) | 60 (20, 180) | 24.5 (5, 365) | 4.54 | 0.103 |
